# Superhuman performance on urology board questions using an explainable language model enhanced with European Association of Urology guidelines

**DOI:** 10.1016/j.esmorw.2024.100078

**Published:** 2024-10-04

**Authors:** M.J. Hetz, N. Carl, S. Haggenmüller, C. Wies, J.N. Kather, M.S. Michel, F. Wessels, T.J. Brinker

**Affiliations:** 1 Digital Biomarkers for Oncology Group, German Cancer Research Center (DKFZ), Heidelberg; 2 Department of Urology, University Medical Center Mannheim, Ruprecht-Karls University of Heidelberg, Mannheim; 3 Medical Faculty, University of Heidelberg, Heidelberg; 4 Else Kroener Fresenius Center for Digital Health, Faculty of Medicine and University Hospital Carl Gustav Carus, TUD Dresden University of Technology, Dresden; 5 Department of Medicine I, Faculty of Medicine and University Hospital Carl Gustav Carus, TUD Dresden University of Technology, Dresden; 6 Medical Oncology Division, National Center for Tumor Diseases (NCT), University Hospital Heidelberg, Heidelberg, Germany

**Keywords:** large language models, evidence-based urology, retrieval augmented generation

## Abstract

**Background:**

Large language models encode clinical knowledge and can answer medical expert questions out-of-the-box without further training. However, this zero-shot performance is limited by outdated training data and lack of explainability impeding clinical translation. We aimed to develop a urology-specialized chatbot (UroBot) and evaluate it against state-of-the-art models as well as historical urologists’ performance in answering urological board questions in a fully clinician-verifiable manner.

**Materials and methods:**

We developed UroBot, a software pipeline based on the GPT-3.5, GPT-4, and GPT-4o models by OpenAI, utilizing retrieval augmented generation and the 2023 European Association of Urology guidelines. UroBot was benchmarked against the zero-shot performance of GPT-3.5, GPT-4, GPT-4o, and Uro_Chat. The evaluation involved 10 runs with 200 European Board of Urology in-service assessment questions, with the performance measured by the mean rate of correct answers (RoCA).

**Results:**

UroBot-4o achieved the highest RoCA, with an average of 88.4%, outperforming GPT-4o (77.6%) by 10.8%. Besides, it is clinician-verifiable and demonstrated the highest level of agreement between runs as measured by Fleiss’ kappa (κ = 0.979). In comparison, the average performance of urologists on urological board questions is 68.7% as reported by the literature.

**Conclusions:**

UroBot is a clinician-verifiable and accurate software pipeline and outperforms published models and urologists in answering urology board questions. We provide code and instructions to use and extend UroBot for further development.

## Highlights

• UroBot achieved clinician-verifiable superhuman performance in medical question answering.
• UroBot surpassed top off-the-shelf language models and urologists, with 88.4% correctness in a board exam.
• Demonstrated superior consistency compared with other off-the-shelf language models.
• UroBot highlights the enhancement of language models with evidence-based knowledge using retrieval augmented generation.
• Code and instructions for UroBot are publicly available for further development.

## Introduction

Researchers are exploring the potential of large language models (LLMs) to tackle medical queries, a frontier that promises to extend how knowledge is accessed in healthcare.^1^, ^2^, ^3^ LLMs are artificial neural networks comprising billions of parameters, trained with a broad spectrum of texts mainly sourced from the internet, which includes medical text sources.^3^, ^4^, ^5^ A recent study assessed the performance of multiple LLMs in answering over 2000 oncological multiple-choice questions. The LLM GPT-4 by OpenAI (San Francisco, CA) achieved the highest rate of correct answers (RoCAs) with 68.7%.^6^ The growing interest across medical specialties in utilizing LLMs for medical question answering (medQA) is culminating in the performance evaluation of LLMs in written medical examinations.^3^^,^^7^, ^8^, ^9^, ^10^ The direct use of LLMs without any further training or context is referred to as a “zero-shot” application.^11^ Although LLMs demonstrate remarkable performance in zero-shot applications, their capabilities are constrained by the training data used, which can be wrong or outdated rapidly. The performances presented by Rydzewski et al.^6^ and other studies evaluating LLM performance in medQA show impressive but in total limited capabilities.^2^, ^3^, ^4^, ^5^, ^6^, ^7^ If LLMs would be used for medical purposes, their accuracy, reliability, and explainability become critical, as these models could significantly impact healthcare decisions, diagnosis, and treatment.^3^

It is, however, possible to augment commercial or open-source LLMs to increase the performance by fine-tuning methods or using retrieval augmented generation (RAG).^12^, ^13^, ^14^ This has led Khene et al.^14^ to adopt an LLM based on evidence-based knowledge with the aim of improving performance. This so-called Uro_Chat was based on GPT-3.5 turbo^15^ using RAG to implement the uro-oncological guidelines published by the European Association of Urology (EAU).^14^^,^^16^ In a subsequent performance test, Uro_Chat was able to answer 61 out of 100 in-service assessment questions (ISA) of the European Board of Urology (EBU) correctly which are integrated into board exams, for example, in Austria, Switzerland, and the Netherlands and thus, would have barely passed a fictitious EBU examination.^17^^,^^18^ Notably, a performance evaluation of GPT-3.5, GPT-4, and Bing AI (now Copilot^19^) using 100 EBU ISA questions revealed 58%-62%, 63%-77%, and 73%-81% correct answers.^8^ The average urologist completes the ISA of the EBU with a grade of 68.7% (standard deviation 6.62).^20^ These results suggest that Uro_Chat performs comparably to GPT-3.5 but is less effective than GPT-4, Copilot, or the average human ISA participant. Nonetheless, Uro_Chat presented an interesting approach by incorporating evidence-based knowledge from guidelines into its design. Similarly, other studies such as a recent study by Ferber et al.,^13^ have demonstrated that the use of RAG improves information retrieval from medical oncology guidelines in gastrointestinal cancer.

Although these developments are exciting, LLMs suffer from the so-called hallucination problem, which describes a phenomenon where the model generates text that is incorrect, nonsensical, or not real. Accordingly, both the European General Data Protection Regulation and clinicians demand explainability of artificial intelligence (AI) for end users, ensuring verifiability of decisions, especially in clinical settings.^21^^,^^22^ Thus development should involve continual collaborations between AI developers and clinical end users.^23^, ^24^, ^25^

Conclusively, the objective of this study is to develop and evaluate an explainable urology-specialized chatbot based on current EAU guidelines against the zero-shot application of state-of-the-art models and urologists’ performance in answering urological board questions in a clinician-verifiable manner.

The design of UroBot incorporates all 2023 guidelines published by the EAU and is engineered to display on which parts of the corresponding documents its answer was based. UroBot’s accuracy is benchmarked against the most recent LLMs, including GPT-3.5, GPT-4, and GPT-4o. Uro_Chat is rebenchmarked to provide a direct comparison to our optimized model. We investigate whether a substantial enhancement is achievable compared with the currently most accurate LLM (GPT-4o). A technical approach to auto-update its knowledge database for its context-based decisions is introduced. All code is made available and instructions are provided for the full reproducibility of our study.

## Material and methods

We adhered to the minimum information about clinical AI modeling documentation standard (MI-CLAIM).^26^

### Material

The EAU guidelines were downloaded as PDF files from the EAU’s online resources on 12 March 2024.^27^ The EAU guidelines were selected due to the comprehensive spectrum of clinically relevant uro-oncologic and urologic evidence-based knowledge, as well as the availability and uniform structure of the text files. In total, the 20 PDF files contained >2000 pages of text. The raw text was extracted from the PDA files using Python (Python Foundation, Wilmington, DE) and split into text chunks with a size of ∼1000 characters, with each chunk tagged with metadata indicating whether it is a paragraph or a table and the corresponding page. Following the segmentation of the text into discrete chunks, the data were transformed into vector embeddings via the open-source embedding model ‘mxbai-embed-large-v1’ by Mixedbread-ai,^28^ with the resulting vectors being stored in a Chroma database.^29^ The instructions and Python code for running UroBot and reproducing our experiments are available via GitHub (https://github.com/DBO-DKFZ/UroBot).^30^ A more detailed description (including Supplementary Tables S1 and S2, available at https://doi.org/10.1016/j.esmorw.2024.100078 for the presentation of data) of the text extraction pipeline can be found in the Supplementary Material, available at https://doi.org/10.1016/j.esmorw.2024.100078.

To assess the performance of UroBot and the competing models under investigation, 200 multiple-choice questions provided by the EBU Committee were digitized by transferring them to an Excel spreadsheet (Microsoft, Redmond, WA). The ISA EBU questions are confidential and are only obtainable through purchase on the official EBU website.^17^

### Question answering pipeline

**Figure 1:**
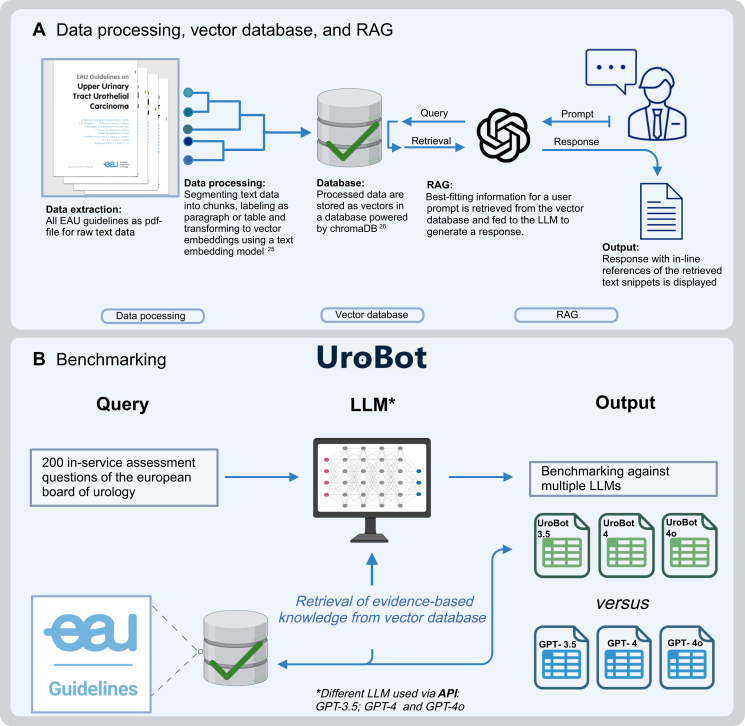
**Design of UroBot and the benchmarking procedure.** (A) The process of the software pipeline creation of UroBot which was separated into several steps, including data extraction, data processing, database creation, and RAG. After segmentation of all raw text data received from all 20 EAU guidelines, a vector database was created by embedding full-text information labeled with paragraphs and tables. RAG comprises the process that is initiated with a user prompt, which first retrieves information from the vector database to generate a response that is then displayed as output alongside in-line references of the retrieved text snippets for user explainability. The time from prompt to response is <5 s. (B) The benchmarking process using 200 ISA EBU questions to test UroBot against all models under investigation (i.e. GPT-3.5, GPT-4, and GPT-4o). API, application programming interface; EAU, European Association of Urology; EBU, European Board of Urology; ISA, in-service assessment; RAG, retrieval augmented generation; LLM, large language model.

To answer the questions, the OpenAI text generation API was used with the models ‘gpt-3.5-turbo-0125’, ‘gpt-4-turbo-2024-04-09’, and ‘gpt-4o-2024-05-13’ (referred to as ‘GPT-3.5’, ‘GPT-4’, and ‘GPT-4o’). Subsequently, RAG was used to make the models urology-informed, leading to the development of UroBot-3.5, UroBot-4, and UroBot-4o based on the respective LLM. To provide UroBot with the necessary context, the query was vectorized using the embedding model described in the preceding text. Similar vectors and their corresponding text chunks are retrieved from the database. The system prompt of UroBot is then modified to contain the retrieved context and prompted to answer the question based on the retrieved content. A visual representation is illustrated in Figure 1. The exact prompts utilized in this study can be accessed in the Supplementary Material, available at https://doi.org/10.1016/j.esmorw.2024.100078. In all experiments, the number of retrieved chunks was set to 10 and the sampling temperature to 0.1. The sampling temperature in a text generation model is a factor in determining the randomness of the generated text. A lower temperature (close to 0) results in a more focused and predictable output, with the selection of words tending toward the most probable.

**Figure 2:**
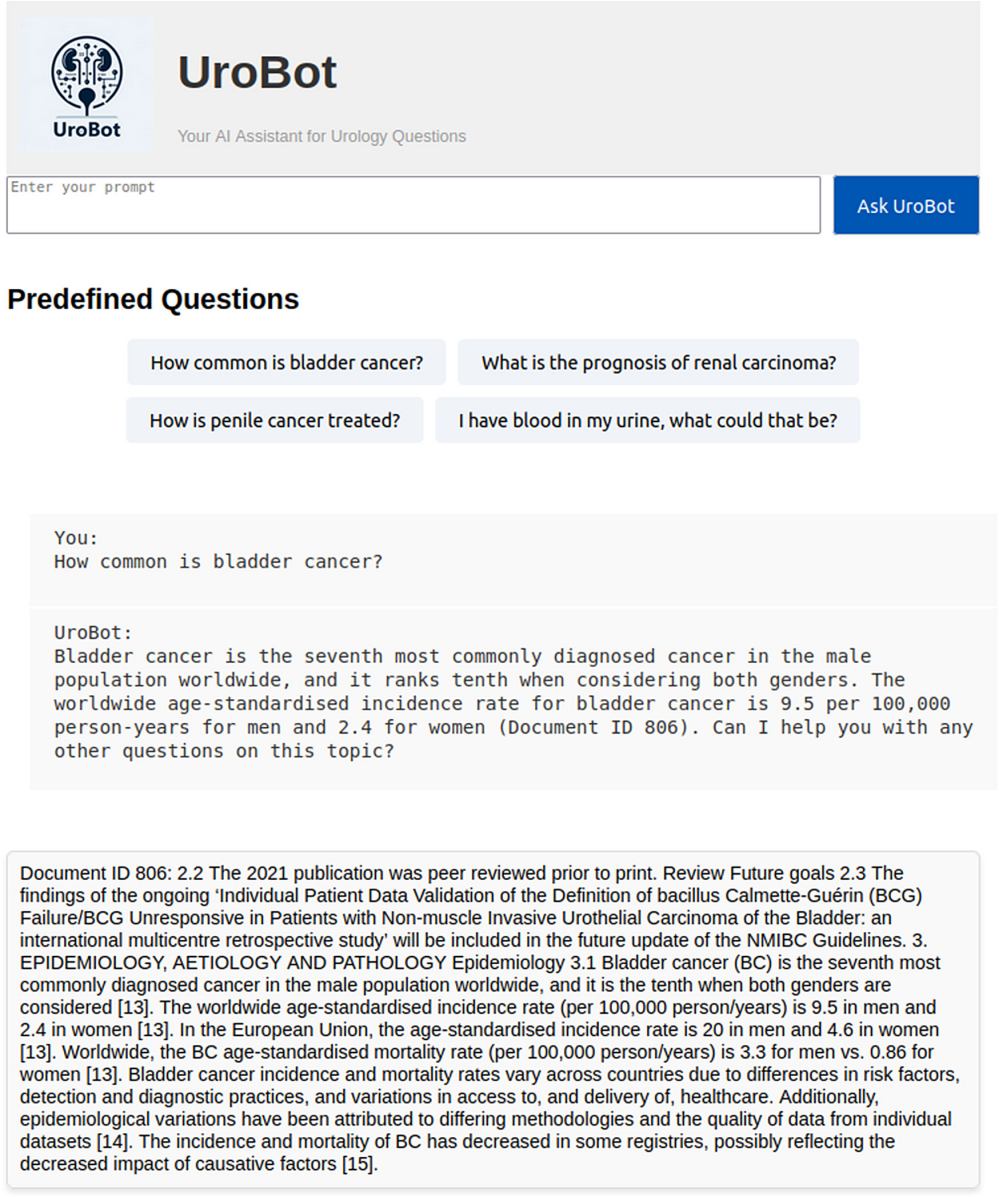
**Screenshot of the user interface of UroBot.** UroBot can be accessed via a web-interfacing application through which users may pose queries and receive responses that are aligned with the guidelines established by the European Association of Urology. UroBot has the capacity to extract the most pertinent information from the database and process it in accordance with user input. Subsequently, the responses are labeled with the references of the text chunks or tables that were utilized, and displayed for the purpose of enhancing transparency. To more effectively demonstrate the capabilities of UroBot, an open-ended question was used in the investigation.

We provided the design of the end-user interface via GitHub.^29^ It displays a query and respective answer with the exact document and text snippet where the information was received from to provide clinician-verifiable LLM outputs (Figure 2).

**Figure 3:**
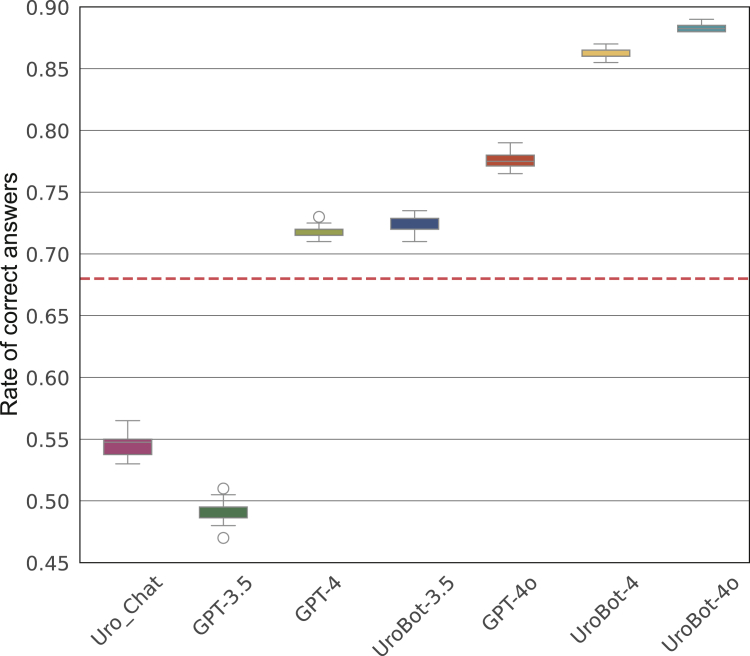
**Boxplots illustrating the rate of correct answers for all models under investigation.** The plot demonstrates that UroBot-4o significantly outperforms all other methods, with a lower variance than the other methods. The dashed line represents the mean performance of urologists as reported in the literature.

### Evaluation

A total of 200 ISA EBU questions were posed to all models. To analyze the consistency of the different models, we repeated this procedure 10 times. The mean RoCA, including 95% confidence intervals (CIs), was used as a performance metric and is calculated by dividing the number of correct answers per run by the total number of questions, averaged over 10 runs. With regard to the OpenAI-based models, an automated benchmark was conducted utilizing the text generation API of OpenAI, without the use of a chat history. With regard to Uro_Chat, all questions were entered into the provided web interface in a consecutive manner 10 times, with the responses then being entered into an Excel spreadsheet. Upon the presentation of each new question, the web interface of Uro_Chat was reloaded.

Fleiss’ kappa was used to evaluate the consistency of the LLM answers, while simultaneously accounting for any agreement that might occur by chance. Fleiss’ kappa ranges from –1 to 1, with 1 representing perfect agreement, 0 denoting agreement expected by chance, and –1 indicating perfect disagreement. Therefore higher values indicate a higher degree of agreement between runs. For statistical comparisons of the LLM performance across all 200 questions, pairwise two-sided *t*-tests were applied. A significance level of alpha 0 .05 was set for all analyses. Significance levels were adjusted to 0.005 (*m* = 10) according to Bonferroni correction^31^ in case of multiple tests to adjust for the increased risk of type I errors due to multiple comparisons.

## Results

### Performance of the models under investigation

An overview of the results is provided in Table 1. The highest RoCA was reached by UroBot-4o with an average of 0.884 (95% CI 0.881-0.886). The highest mean RoCA of a standard model (without RAG) was reached by GPT-4o with 0.776 (95% CI 0.771-0.781). UroBot-4o outperformed the best standard model by a Δ of 0.108 pairwise two-sided *t*-test (*P* < 0.001; see Table 1). The performance of UroBot was dependent on the LLM used. The mean RoCA was 0.722 (95% CI 0.717-0.728) for UroBot-3.5, 0.863 (95% CI 0.860-0.867) for UroBot-4, and 0.884 (95% CI 0.881-0.886) for UroBot-4o.

**Table 1:** Benchmark results for all models aggregated over 10 runs

Benchmark	Uro_Chat	GPT-3.5	GPT-4	UroBot-3.5	GPT-4o	UroBot-4	UroBot-4o
Mean rate of correct answers (95% confidence interval)	0.547 (0.538-0.555)	0.492 (0.484-0.500)	0.719 (0.715-0.723)	0.722 (0.717-0.728)	0.776 (0.771-0.781)	0.863 (0.860-0.867)	0.884 (0.881-0.886)
Majority voting rate of correct answers	0.57	0.5	0.72	0.715	0.78	0.865	0.885
*P* value (versus UroBot-4o)	<0.001	<0.001	<0.001	<0.001	<0.001	<0.001	Ref.
Fleiss’ kappa value	0.704	0.866	0.945	0.92	0.943	0.966	0.979

The results demonstrate that UroBot-4o exhibits a markedly higher rate of correct answers than its competitors. Furthermore, it has the highest kappa value, indicating the highest consistency in answering. An overview of the results is provided in Figure 3.

The lowest performance was observed for Uro_Chat with an RoCA of 0.547 (95% CI 0.538-0.555) and GPT-3.5 turbo with 0.492 (95% CI 0.484-0.500).

### Consistency of performance across test runs

The reliability of the generated model answers was assessed by presenting the same question to various models on 10 separate occasions. In general, the agreement between test runs was *substantial* across all LLMs and test runs according to the interpretation guideline of Landis and Koch.^32^ UroBot-4o showed the highest agreement, demonstrating almost perfect consistency between test runs (κ = 0.979), followed by UroBot-4 (κ = 0.966) and GPT-4 (κ = 0.945). The lowest agreement was observed with Uro_Chat, which nevertheless showed substantial agreement between test runs (κ = 0.70).

## Discussion

In this study, we developed UroBot, an RAG-enhanced LLM capable of answering questions based on text information from a vector database containing all 20 available EAU guidelines. Our findings demonstrate that UroBot exhibits superior performance in urological board question answering, achieving an average RoCA of 88.4%. This performance not only surpassed the zero-shot GPT-4o model by a margin of 10.8 percentage points, but also greatly exceeded the average performance of urologists on board questions, which is reported at 68.7% in the literature.^20^ Furthermore, UroBot-4o’s outputs were clinician-verifiable, ensuring that the responses align with medical standards. The model exhibited the highest level of consistency across multiple runs, as evidenced by a Fleiss’ kappa value of 0.979, indicating almost perfect agreement. The retrieval mechanism used by RAG is crucial in providing contextually appropriate information, which the LLM then effectively utilizes to produce accurate answers. Results of the benchmarking show that UroBot significantly outperforms the best available models, surpassing previously reported performance levels in the literature and the average ISA attendee’s performance.^6^^,^^8^^,^^18^^,^^20^ The lowest results for correctness and consistency were observed in Uro_Chat and GPT-3.5 turbo.

While off-the-shelf LLMs demonstrate impressive capabilities in medQA, a substantial limitation is the insufficient performance.^6^^,^^10^ This constraint can be mitigated through in-context learning, for example, via prompt engineering. However, this method is hindered by the fixed length of the input string, which can accommodate only a limited number of pages of text data, rendering this approach impractical.^11^ RAG is an advanced form of in-context learning that can utilize extensive knowledge bases. Unlike traditional in-context learning, RAG incorporates an external knowledge retrieval system. Recent models from other AI research groups have also demonstrated impressive improvements using RAG, consistent with the success observed in our study. Ferber et al.^13^ achieved 84% correct statements in a subset of medical oncology questions with an RAG-enhanced model compared with 57% with the standard model.^13^

It is of critical importance to embed medical knowledge into a model, particularly in rapidly evolving fields such as urology, where guidelines are frequently updated. RAG offers scalability and easy-to-implement updates, which provides a method for maintaining current and evidence-based assistance tools in patient care and could therefore benefit clinicians as an informational or educational tool. Our study demonstrates significant performance improvements using a feasible way to incorporate evidence-based knowledge into LLMs using the RAG method. Importantly, RAG might be the key to paving the way for clinically useful LLMs.

### Limitations

Although leveraging medical state-of-the-art training material for the EBU examination exclusively, the reliance on 200 multiple-choice questions from the EBU Committee may not be fully representative of the full range of scenarios in clinical practice. Future research could build upon this work by testing UroBot with additional questions and clinical situations, allowing practitioners to interact with UroBot in daily tasks.

It is recommended that open-ended questions are included in future assessments to further evaluate the reasoning abilities of UroBot. Furthermore, this study does not investigate the effects of different prompts on performance. Future research should explore this topic, particularly focusing on the potential brittleness^33^ of prompts and the model’s robustness to variations in statements. It is crucial to understand how changes in prompt phrasing might affect the responses generated by UroBot, as this could impact its reliability in diverse real-world situations.^34^

Further, a urologist in a board examination does not have all 20 EAU guidelines available and can retrieve data from them, as our model did. If time permits and a urologist had 10 h per board question to search through thousands of pages of guidelines, they might even achieve 100% accuracy on the board questions. Nonetheless, UroBot gives precise and verifiable answers within <5 s. In terms of speed *and* accuracy, UroBot’s performance is superhuman.

Although RAG is effective in reducing the occurrence of hallucinations, it does not entirely prevent them.^35^^,^^36^ LLMs may still utilize information outside the provided context to answer the question. For clinical use, a mechanism for detecting hallucinations may be necessary. Furthermore, the retrieved text data may exhibit a high degree of textual similarity to the query, yet may lack the necessary relevance to answer the question. We used a commercial LLM as a backbone for RAG (ChatGPT-4o), nonetheless, open-source architectures of comparable performance are also available (e.g. LLAMA-3 by Meta, Menlo Park, CA).

In addition, if LLMs were to be used as an information source or if the decision-making process of clinicians would be influenced, they must be approved as a medical device.^37^^,^^38^ In our ongoing research, we are evaluating open-ended prompts and will develop a user-friendly interface displaying outputs similar to EAU guidelines. UroBot will feature distinct physician and patient user modes, tailored to specific needs, to ensure effectiveness and safety. This approach aims to enhance the consistency and accuracy of LLMs in clinical applications, providing reliable AI-assistance tools incorporating evidence-based knowledge and individual patient data at the same time. These improvements act as the cornerstone for further clinical research.

## Conclusion

This study highlights the potential of enhancing LLMs with evidence-based guidelines to improve their performance in specialized medical fields. UroBot is clinician-verifiable and substantially more accurate as compared with both the performance of published models and urologists in answering board questions, encouraging translation to care and showcasing the benefit of RAG. We provide code and instructions to rebuild UroBot and its user interface for further development. As we further refine these models and expand their knowledge bases, the integration of LLMs into routine medical practice becomes an increasingly viable and beneficial prospect. UroBot will be developed as an information system, designed to assist in navigating the landscape of evidence-based medical knowledge, particularly by leveraging the EAU guidelines as a reference for reliable, up-to-date, accurate, evidence-based, and foremost user-verifiable information. In our ongoing research, we are investigating open-ended prompts and developing a user-friendly interface that presents outputs similar to the EAU guidelines to conclude the preclinical phase of our project. In accordance with the DECIDE-AI^39^ guidelines, upon successful completion of the preclinical phase, we plan to involve urologists in early live clinical evaluations to test safety and effectiveness. Nonetheless, regulatory approval as medical devices is imperative to all LLMs before their implementation into individual patient care.^37^ In addition, the current software is still unable to replace doctor–patient relationships and can and should not take responsibility for medical decisions because many patients would be opposed to this, especially in oncologic settings.^22^
