# Corrigendum to ‘Superhuman performance on urology board questions using an explainable language model enhanced with European Association of Urology guidelines’ [ESMO Real World Data and Digital Oncology, Volume 6, December 2024, 100078]

**DOI:** 10.1016/j.esmorw.2026.100687

**Published:** 2026-02-17

**Authors:** M.J. Hetz, N. Carl, S. Haggenmüller, C. Wies, J.N. Kather, M.S. Michel, F. Wessels, T.J. Brinker

**Affiliations:** 1 Digital Biomarkers for Oncology Group, German Cancer Research Center (DKFZ), Heidelberg; 2 Department of Urology, University Medical Center Mannheim, Ruprecht-Karls University of Heidelberg, Mannheim; 3 Medical Faculty, University of Heidelberg, Heidelberg; 4 Else Kroener Fresenius Center for Digital Health, Faculty of Medicine and University Hospital Carl Gustav Carus, TUD Dresden University of Technology, Dresden; 5 Department of Medicine I, Faculty of Medicine and University Hospital Carl Gustav Carus, TUD Dresden University of Technology, Dresden; 6 Medical Oncology Division, National Center for Tumor Diseases (NCT), University Hospital Heidelberg, Heidelberg, Germany

The authors regret that the original article did not explicitly state that the 2024 European Association of Urology (EAU) Guidelines were downloaded and utilised with written permission from the EAU Guidelines Office. The guidelines were used as a reference source to support the development and evaluation of the language model. The study was conducted independently, and responsibility for the content, interpretation, and conclusions of the work lies solely with the authors. The EAU was not involved in the study design, analysis, or manuscript preparation and does not endorse or assume responsibility for the application or its outputs. In the event of any discrepancies, the original EAU Guidelines remain the authoritative source.

The authors would like to apologise for any inconvenience caused.

