# Supplementary material for: Superhuman performance on urology board questions using an explainable language model enhanced with European Association of Urology guidelines
Source: ESMO Real World Data Digit Oncol. 2024 Oct 4;6:100078. doi: 10.1016/j.esmorw.2024.100078 (PMC12836625; doi:10.1016/j.esmorw.2024.100078)
Supplement: Supplementary Data [file mmc1.docx]

**Supplementary Material for the Manuscript:**

**Title:** Superhuman Performance on Urology Board Questions Using an Explainable Language Model Enhanced with European Association of Urology Guidelines

The full code is available via Github under: <https://github.com/DBO-DKFZ/UroBot> or using the QR-Code below:

**
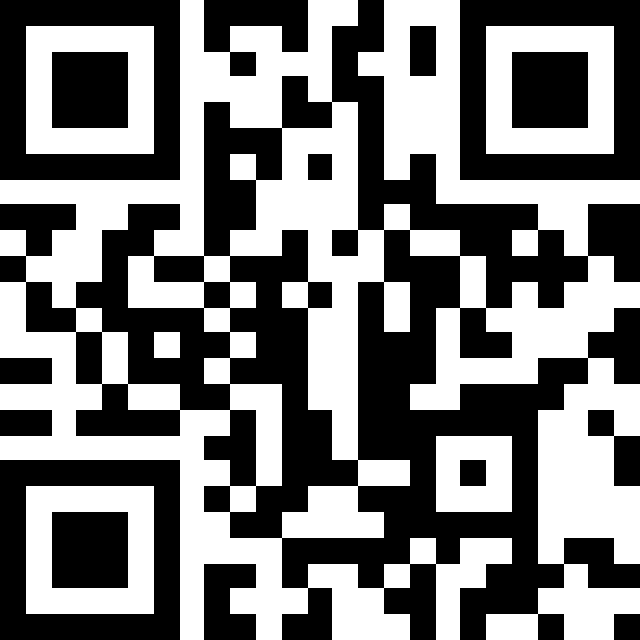
**

**Supplementary Methods:**

**Text Extraction Pipeline**

In order to utilise the information from the PDF files, it is necessary to extract the text and tables from the individual PDF files and store the text chunks in a vector database. A variety of Python libraries are employed for the purpose of extracting data from PDF files, including PyMuPDF^1^, Camelot^2^ and PDFMiner^3^. The aforementioned software applications differ from one another in terms of their functionality and capabilities. For instance, they vary in their ability to extract data from tables.

The initial stage of the process is to read the document and then divide it into discrete text segments. The data was divided into paragraphs and subsequently combined into larger units, with each unit comprising approximately 1,000 characters. This approach was taken in order to avoid disrupting complete sentences, taking into account line ending characters (such as the full stop, exclamation mark and question mark).

The subsequent stage of the extraction pipeline entails the removal of all extraneous material, namely that which precedes the Introduction section and that which follows the References section. This is because such material introduces irrelevant text data into the database. This is achieved by utilising regular expressions to search for the respective patterns, such as References or Introduction. The subsequent stage is the extraction of tables, which are essential for the aforementioned EAU guidelines.

The process of identifying captions of tables begins with the application of regular expressions and a search for a combination of patterns, such as 'Table\s+\d+:' or 'Table\s+\d+\.\d+'. The page number and y-coordinate are stored for subsequent matching of the extracted captions and the corresponding table. The matching process involves a comparison of the coordinates of the identified tables and the captions extracted from them. In instances where tables are distributed across multiple pages, they are merged to form a single table. The captions are then incorporated as the top row of the extracted tables, providing additional context for the tables.

The final step involves the removal of line breaks from the tables and their transformation into a Markdown table format. This facilitates the interpretation of the tables by language models. Once all textual information has been extracted, the vector database can be built.

**Vector database**

The vector database is built using a locally running ChromaDB^4^ client and a custom embedding function that uses the open source embedding model 'mxbai-embed-large-v1' from Mixedbread-ai. This embedding model was chosen based on its high ranking in the 'Massive Text Embedding Benchmark (MTEB)'^5^ available on Hugging Face and its comparatively small number of parameters. An embedding model with a smaller number of parameters can be run at high speed on most laptops, making it easier to run UroBot on a wide range of devices.

All available text chunks and tables are iterated over and added to the database. Each item is assigned a document ID, a data type (text or table), the name of the original file, the page number, and the vector obtained by applying the embedding model to each text chunk or table. The embedding model is applied using the Sentence Transformers^6^ Python library, which allows for the rapid and straightforward utilisation of a diverse range of text embedding models.

**Querying UroBot**

The process by which a query is transformed into an answer by UroBot is as follows:

1. Vectorising the user query
2. A comparison is then made between the vectorised query and the vectorised text chunks in the database, with the 10 most fitting text chunks being retrieved.
3. The retrieved text chunks, including the document ID, are incorporated into the system prompt, as illustrated in the section on used prompts in the supplementary materials.
4. Calling the OpenAI "chat completion" Application Programming Interface (API) in order to elicit a response from the respective model.

**Benchmarking procedure**

In order to facilitate the benchmarking process, we have automated the process of inferring the responses of the various models to the benchmark questions. The 200 questions contained in the Excel spreadsheet were then iterated over in their entirety. As can be observed in the system prompts employed, the model is prompted to respond with a character that resembles the correct answer. However, this approach is not fail-safe. The models occasionally employed quotation marks in their responses, whereas on other occasions they did not. In a small number of instances, they provided a sentence that indicated the correct answer. Consequently, we implemented basic string filtering techniques to extract solely the character that signified the response. All outcomes were stored in a CSV file. Following the benchmarking process, the CSV file was manually examined to ascertain that the responses were in the appropriate format and that any formatting issues, such as the presence of multiple characters rather than a single one, had been rectified. Subsequently, the rate of correct answers (RoCA) was calculated for each model using the following formula:

$$RoCA= \frac{Number of correct answers}{Number of total answers}$$

1: <https://pymupdf.readthedocs.io/>

2: <https://camelot-py.readthedocs.io/>

3: <https://pdfminersix.readthedocs.io>
4: https://www.trychroma.com/
5: <https://huggingface.co/spaces/mteb/leaderboard>
6: https://sbert.net/

**Supplementary Results:**

**Table 1:** Summarised results of the different models over ten runs.

| **model** | **mean_roca** | **majority_vote_roca** | **1** | **2** | **3** | **4** | **5** | **6** | **7** | **8** | **9** | **10** |
| --- | --- | --- | --- | --- | --- | --- | --- | --- | --- | --- | --- | --- |
| Uro_Chat | 0.547 | 0.57 | 0.55 | 0.565 | 0.53 | 0.55 | 0.545 | 0.53 | 0.545 | 0.535 | 0.565 | 0.55 |
| GPT_3.5 | 0.492 | 0.5 | 0.495 | 0.48 | 0.495 | 0.495 | 0.49 | 0.505 | 0.495 | 0.51 | 0.485 | 0.47 |
| GPT_4 | 0.719 | 0.72 | 0.72 | 0.72 | 0.72 | 0.73 | 0.725 | 0.715 | 0.715 | 0.72 | 0.715 | 0.71 |
| UroGPT_3.5 | 0.722 | 0.715 | 0.73 | 0.735 | 0.72 | 0.725 | 0.72 | 0.72 | 0.715 | 0.73 | 0.72 | 0.71 |
| UroGPT_4 | 0.863 | 0.865 | 0.865 | 0.87 | 0.865 | 0.865 | 0.865 | 0.87 | 0.86 | 0.86 | 0.855 | 0.86 |
| GPT_4o | 0.776 | 0.78 | 0.78 | 0.77 | 0.775 | 0.79 | 0.775 | 0.77 | 0.775 | 0.78 | 0.765 | 0.78 |
| **UroGPT_4o** | **0.883** | **0.885** | **0.89** | **0.885** | **0.885** | **0.88** | **0.88** | **0.89** | **0.885** | **0.88** | **0.88** | **0.88** |

**Table 2:** Each Spreadsheet represents one LLM with its respective test runs. Runs 1 through 10 are displayed in the columns. The rows represent the Question ID, displayed in the same sequence as in the MCQ ISA booklet 2021-2022. The observations represent correct (1) or incorrect (0) answers. For further reading please address the MCQ ISA booklet. The questions are under copyright and not publicly available. They can be purchased on [www.ebu.com](http://www.ebu.com).

| **ID** | **Uro_Chat_1** | **Uro_Chat_2** | **Uro_Chat_3** | **Uro_Chat_4** | **Uro_Chat_5** | **Uro_Chat_6** | **Uro_Chat_7** | **Uro_Chat_8** | **Uro_Chat_9** | **Uro_Chat_10** |
| --- | --- | --- | --- | --- | --- | --- | --- | --- | --- | --- |
| 1 | 1 | 0 | 0 | 0 | 0 | 0 | 0 | 0 | 0 | 0 |
| 2 | 0 | 0 | 0 | 1 | 1 | 0 | 0 | 0 | 0 | 0 |
| 3 | 0 | 0 | 0 | 1 | 1 | 1 | 1 | 1 | 1 | 1 |
| 4 | 0 | 0 | 0 | 0 | 1 | 0 | 0 | 0 | 0 | 1 |
| 5 | 1 | 1 | 1 | 1 | 1 | 1 | 1 | 1 | 1 | 1 |
| 6 | 1 | 1 | 1 | 1 | 1 | 1 | 1 | 1 | 1 | 1 |
| 7 | 1 | 1 | 1 | 1 | 1 | 1 | 1 | 1 | 1 | 1 |
| 8 | 1 | 0 | 1 | 1 | 1 | 1 | 1 | 1 | 1 | 1 |
| 9 | 1 | 1 | 1 | 1 | 1 | 1 | 1 | 1 | 1 | 1 |
| 10 | 0 | 0 | 0 | 1 | 1 | 1 | 1 | 1 | 1 | 1 |
| 11 | 0 | 0 | 0 | 0 | 0 | 0 | 0 | 0 | 0 | 0 |
| 12 | 1 | 1 | 1 | 1 | 1 | 0 | 0 | 0 | 0 | 0 |
| 13 | 0 | 0 | 0 | 1 | 1 | 1 | 1 | 1 | 1 | 1 |
| 14 | 1 | 1 | 1 | 1 | 1 | 1 | 1 | 1 | 1 | 1 |
| 15 | 1 | 1 | 1 | 1 | 1 | 1 | 1 | 1 | 1 | 1 |
| 16 | 0 | 0 | 0 | 0 | 0 | 0 | 0 | 0 | 0 | 0 |
| 17 | 1 | 1 | 1 | 1 | 1 | 1 | 1 | 1 | 1 | 1 |
| 18 | 0 | 0 | 0 | 0 | 0 | 0 | 0 | 0 | 0 | 0 |
| 19 | 0 | 0 | 0 | 0 | 0 | 0 | 0 | 0 | 0 | 0 |
| 20 | 0 | 0 | 0 | 0 | 0 | 0 | 0 | 0 | 0 | 0 |
| 21 | 0 | 0 | 0 | 1 | 1 | 1 | 1 | 1 | 1 | 1 |
| 22 | 0 | 1 | 0 | 0 | 0 | 0 | 0 | 0 | 0 | 0 |
| 23 | 1 | 0 | 0 | 1 | 1 | 1 | 1 | 1 | 1 | 1 |
| 24 | 0 | 0 | 0 | 0 | 0 | 0 | 0 | 0 | 0 | 0 |
| 25 | 1 | 0 | 0 | 1 | 1 | 1 | 1 | 1 | 1 | 1 |
| 26 | 0 | 0 | 0 | 0 | 1 | 0 | 0 | 0 | 0 | 1 |
| 27 | 0 | 0 | 0 | 0 | 0 | 0 | 0 | 0 | 0 | 0 |
| 28 | 1 | 1 | 1 | 1 | 1 | 1 | 1 | 1 | 1 | 1 |
| 29 | 1 | 1 | 1 | 1 | 1 | 1 | 1 | 1 | 1 | 1 |
| 30 | 0 | 0 | 0 | 1 | 1 | 1 | 1 | 1 | 1 | 1 |
| 31 | 1 | 0 | 0 | 0 | 0 | 0 | 0 | 0 | 0 | 0 |
| 32 | 0 | 0 | 0 | 1 | 0 | 0 | 0 | 1 | 1 | 0 |
| 33 | 1 | 1 | 1 | 1 | 1 | 1 | 1 | 1 | 1 | 1 |
| 34 | 1 | 1 | 1 | 1 | 1 | 1 | 1 | 1 | 1 | 1 |
| 35 | 0 | 1 | 1 | 0 | 0 | 0 | 0 | 0 | 0 | 0 |
| 36 | 1 | 1 | 1 | 0 | 0 | 0 | 1 | 0 | 0 | 0 |
| 37 | 0 | 0 | 0 | 0 | 0 | 0 | 0 | 0 | 0 | 0 |
| 38 | 0 | 0 | 0 | 0 | 0 | 0 | 0 | 0 | 0 | 0 |
| 39 | 0 | 1 | 1 | 0 | 1 | 0 | 0 | 0 | 0 | 0 |
| 40 | 0 | 0 | 0 | 1 | 0 | 0 | 0 | 0 | 0 | 0 |
| 41 | 0 | 0 | 0 | 0 | 0 | 0 | 0 | 0 | 0 | 0 |
| 42 | 0 | 0 | 0 | 1 | 0 | 0 | 0 | 0 | 0 | 0 |
| 43 | 0 | 0 | 0 | 0 | 0 | 0 | 0 | 0 | 0 | 0 |
| 44 | 1 | 1 | 1 | 1 | 1 | 1 | 1 | 1 | 1 | 1 |
| 45 | 1 | 1 | 1 | 1 | 1 | 1 | 1 | 1 | 1 | 1 |
| 46 | 1 | 1 | 1 | 1 | 1 | 1 | 1 | 1 | 1 | 1 |
| 47 | 1 | 1 | 1 | 1 | 1 | 1 | 1 | 1 | 1 | 1 |
| 48 | 1 | 1 | 1 | 1 | 0 | 0 | 0 | 0 | 0 | 0 |
| 49 | 1 | 1 | 1 | 0 | 1 | 0 | 1 | 0 | 1 | 0 |
| 50 | 1 | 1 | 0 | 0 | 0 | 1 | 0 | 0 | 0 | 0 |
| 51 | 0 | 0 | 0 | 0 | 0 | 0 | 0 | 0 | 0 | 0 |
| 52 | 0 | 0 | 0 | 0 | 0 | 0 | 0 | 0 | 0 | 0 |
| 53 | 0 | 0 | 0 | 0 | 0 | 0 | 0 | 0 | 0 | 0 |
| 54 | 1 | 0 | 1 | 0 | 0 | 0 | 0 | 0 | 0 | 0 |
| 55 | 1 | 1 | 1 | 1 | 1 | 1 | 1 | 1 | 1 | 1 |
| 56 | 0 | 1 | 1 | 1 | 1 | 1 | 1 | 1 | 1 | 1 |
| 57 | 0 | 0 | 0 | 0 | 0 | 0 | 0 | 0 | 0 | 0 |
| 58 | 1 | 1 | 1 | 1 | 1 | 1 | 1 | 1 | 1 | 1 |
| 59 | 1 | 1 | 1 | 1 | 1 | 1 | 1 | 1 | 1 | 1 |
| 60 | 1 | 1 | 1 | 1 | 0 | 1 | 1 | 1 | 1 | 1 |
| 61 | 0 | 0 | 0 | 1 | 1 | 1 | 1 | 1 | 1 | 1 |
| 62 | 1 | 0 | 1 | 0 | 0 | 0 | 0 | 0 | 0 | 0 |
| 63 | 1 | 1 | 1 | 1 | 1 | 1 | 1 | 1 | 1 | 1 |
| 64 | 0 | 0 | 1 | 1 | 1 | 1 | 1 | 1 | 1 | 1 |
| 65 | 0 | 0 | 0 | 0 | 0 | 0 | 0 | 0 | 0 | 0 |
| 66 | 1 | 1 | 1 | 1 | 1 | 1 | 1 | 1 | 1 | 1 |
| 67 | 1 | 1 | 1 | 1 | 1 | 1 | 1 | 1 | 1 | 1 |
| 68 | 1 | 1 | 1 | 1 | 1 | 1 | 1 | 1 | 1 | 1 |
| 69 | 1 | 1 | 1 | 1 | 0 | 1 | 1 | 1 | 1 | 1 |
| 70 | 1 | 1 | 1 | 0 | 1 | 1 | 1 | 1 | 1 | 1 |
| 71 | 1 | 1 | 1 | 1 | 0 | 0 | 1 | 1 | 1 | 1 |
| 72 | 1 | 1 | 1 | 1 | 1 | 1 | 1 | 1 | 1 | 1 |
| 73 | 1 | 1 | 1 | 1 | 1 | 1 | 1 | 1 | 1 | 1 |
| 74 | 1 | 1 | 1 | 1 | 1 | 1 | 1 | 1 | 1 | 1 |
| 75 | 1 | 1 | 1 | 1 | 1 | 1 | 1 | 1 | 1 | 1 |
| 76 | 1 | 1 | 1 | 1 | 1 | 1 | 1 | 1 | 1 | 1 |
| 77 | 0 | 0 | 0 | 0 | 0 | 0 | 0 | 0 | 0 | 0 |
| 78 | 0 | 0 | 0 | 0 | 0 | 0 | 0 | 0 | 0 | 0 |
| 79 | 1 | 1 | 1 | 1 | 1 | 1 | 1 | 1 | 1 | 1 |
| 80 | 0 | 0 | 0 | 0 | 0 | 0 | 0 | 0 | 0 | 0 |
| 81 | 0 | 0 | 0 | 0 | 0 | 0 | 0 | 0 | 0 | 0 |
| 82 | 1 | 1 | 1 | 0 | 0 | 0 | 0 | 0 | 0 | 0 |
| 83 | 1 | 1 | 1 | 1 | 1 | 1 | 1 | 1 | 1 | 1 |
| 84 | 0 | 0 | 0 | 0 | 0 | 0 | 0 | 0 | 0 | 0 |
| 85 | 0 | 0 | 0 | 0 | 0 | 0 | 0 | 0 | 0 | 0 |
| 86 | 1 | 1 | 1 | 0 | 0 | 0 | 0 | 0 | 0 | 0 |
| 87 | 1 | 1 | 0 | 0 | 0 | 0 | 0 | 0 | 0 | 0 |
| 88 | 1 | 1 | 1 | 1 | 1 | 1 | 1 | 1 | 1 | 1 |
| 89 | 1 | 1 | 1 | 1 | 1 | 1 | 1 | 1 | 1 | 1 |
| 90 | 1 | 1 | 1 | 1 | 1 | 1 | 1 | 1 | 1 | 1 |
| 91 | 1 | 1 | 1 | 1 | 1 | 1 | 1 | 1 | 1 | 1 |
| 92 | 1 | 1 | 1 | 1 | 1 | 1 | 1 | 1 | 1 | 1 |
| 93 | 1 | 1 | 1 | 1 | 1 | 1 | 1 | 1 | 1 | 1 |
| 94 | 1 | 1 | 1 | 1 | 1 | 1 | 1 | 1 | 1 | 1 |
| 95 | 1 | 1 | 1 | 1 | 1 | 1 | 1 | 1 | 1 | 1 |
| 96 | 1 | 1 | 1 | 1 | 1 | 1 | 1 | 1 | 1 | 1 |
| 97 | 1 | 1 | 1 | 1 | 1 | 1 | 1 | 1 | 1 | 1 |
| 98 | 0 | 0 | 0 | 0 | 1 | 0 | 0 | 0 | 0 | 0 |
| 99 | 0 | 1 | 0 | 0 | 1 | 1 | 1 | 1 | 1 | 1 |
| 100 | 1 | 1 | 1 | 1 | 0 | 0 | 1 | 1 | 1 | 0 |
| 101 | 0 | 1 | 0 | 1 | 1 | 1 | 1 | 1 | 1 | 1 |
| 102 | 1 | 1 | 1 | 1 | 1 | 1 | 1 | 1 | 1 | 1 |
| 103 | 0 | 0 | 0 | 0 | 0 | 0 | 0 | 0 | 0 | 0 |
| 104 | 0 | 0 | 0 | 1 | 1 | 1 | 1 | 1 | 1 | 1 |
| 105 | 1 | 1 | 1 | 1 | 1 | 1 | 1 | 1 | 1 | 1 |
| 106 | 0 | 0 | 0 | 0 | 0 | 0 | 0 | 0 | 0 | 0 |
| 107 | 0 | 1 | 1 | 1 | 1 | 1 | 1 | 1 | 1 | 1 |
| 108 | 0 | 1 | 1 | 0 | 1 | 1 | 1 | 1 | 1 | 1 |
| 109 | 0 | 0 | 0 | 0 | 0 | 0 | 0 | 0 | 0 | 0 |
| 110 | 1 | 1 | 0 | 0 | 0 | 0 | 0 | 0 | 0 | 0 |
| 111 | 1 | 1 | 1 | 1 | 1 | 1 | 1 | 1 | 1 | 1 |
| 112 | 0 | 0 | 0 | 0 | 0 | 0 | 0 | 0 | 0 | 0 |
| 113 | 1 | 1 | 1 | 1 | 1 | 1 | 1 | 1 | 1 | 1 |
| 114 | 1 | 0 | 1 | 1 | 1 | 1 | 1 | 1 | 1 | 1 |
| 115 | 0 | 0 | 0 | 0 | 0 | 0 | 0 | 0 | 0 | 0 |
| 116 | 1 | 1 | 1 | 0 | 0 | 0 | 0 | 0 | 0 | 0 |
| 117 | 1 | 1 | 1 | 0 | 0 | 0 | 0 | 0 | 0 | 0 |
| 118 | 1 | 1 | 1 | 1 | 1 | 1 | 1 | 1 | 1 | 1 |
| 119 | 1 | 1 | 1 | 1 | 1 | 1 | 1 | 1 | 1 | 1 |
| 120 | 0 | 0 | 0 | 1 | 1 | 1 | 1 | 1 | 1 | 1 |
| 121 | 0 | 1 | 1 | 1 | 0 | 1 | 1 | 1 | 1 | 1 |
| 122 | 0 | 0 | 0 | 1 | 1 | 1 | 1 | 1 | 1 | 1 |
| 123 | 1 | 1 | 1 | 1 | 1 | 1 | 1 | 1 | 1 | 1 |
| 124 | 1 | 1 | 1 | 1 | 1 | 1 | 1 | 1 | 1 | 1 |
| 125 | 1 | 1 | 1 | 1 | 1 | 1 | 1 | 1 | 1 | 1 |
| 126 | 0 | 0 | 0 | 0 | 0 | 0 | 0 | 0 | 0 | 0 |
| 127 | 1 | 1 | 1 | 0 | 0 | 0 | 0 | 0 | 0 | 0 |
| 128 | 1 | 1 | 1 | 1 | 1 | 1 | 1 | 1 | 1 | 1 |
| 129 | 0 | 1 | 0 | 0 | 0 | 0 | 0 | 0 | 0 | 0 |
| 130 | 0 | 0 | 0 | 1 | 1 | 1 | 1 | 1 | 1 | 1 |
| 131 | 0 | 0 | 0 | 0 | 0 | 0 | 0 | 0 | 0 | 0 |
| 132 | 0 | 0 | 0 | 0 | 0 | 0 | 0 | 0 | 0 | 0 |
| 133 | 1 | 1 | 1 | 1 | 1 | 1 | 1 | 1 | 1 | 1 |
| 134 | 1 | 1 | 1 | 1 | 1 | 1 | 1 | 1 | 1 | 1 |
| 135 | 0 | 0 | 0 | 0 | 0 | 0 | 0 | 0 | 0 | 0 |
| 136 | 1 | 1 | 1 | 1 | 1 | 1 | 1 | 1 | 1 | 1 |
| 137 | 0 | 0 | 0 | 0 | 0 | 0 | 0 | 0 | 0 | 0 |
| 138 | 0 | 1 | 0 | 0 | 0 | 0 | 0 | 0 | 0 | 0 |
| 139 | 0 | 0 | 0 | 0 | 0 | 0 | 0 | 0 | 0 | 0 |
| 140 | 0 | 0 | 0 | 1 | 1 | 1 | 0 | 1 | 1 | 1 |
| 141 | 1 | 1 | 1 | 0 | 0 | 0 | 0 | 0 | 0 | 0 |
| 142 | 1 | 1 | 1 | 1 | 1 | 1 | 1 | 1 | 1 | 1 |
| 143 | 1 | 1 | 1 | 1 | 1 | 1 | 1 | 1 | 1 | 1 |
| 144 | 0 | 0 | 0 | 0 | 0 | 0 | 0 | 0 | 0 | 0 |
| 145 | 1 | 1 | 1 | 1 | 1 | 1 | 1 | 1 | 1 | 1 |
| 146 | 0 | 0 | 0 | 0 | 0 | 0 | 0 | 0 | 0 | 0 |
| 147 | 1 | 1 | 1 | 1 | 1 | 1 | 1 | 1 | 1 | 1 |
| 148 | 0 | 0 | 0 | 0 | 0 | 0 | 0 | 0 | 0 | 0 |
| 149 | 0 | 0 | 0 | 0 | 0 | 0 | 0 | 0 | 0 | 0 |
| 150 | 0 | 0 | 0 | 0 | 0 | 0 | 0 | 0 | 0 | 0 |
| 151 | 1 | 0 | 1 | 1 | 1 | 1 | 1 | 1 | 1 | 1 |
| 152 | 0 | 0 | 0 | 0 | 0 | 0 | 0 | 0 | 0 | 0 |
| 153 | 0 | 0 | 0 | 0 | 0 | 0 | 0 | 0 | 0 | 0 |
| 154 | 0 | 0 | 0 | 0 | 0 | 0 | 0 | 0 | 0 | 0 |
| 155 | 1 | 1 | 1 | 1 | 1 | 1 | 1 | 1 | 1 | 1 |
| 156 | 1 | 1 | 1 | 0 | 0 | 0 | 0 | 0 | 1 | 1 |
| 157 | 0 | 0 | 0 | 0 | 0 | 0 | 0 | 0 | 0 | 0 |
| 158 | 0 | 0 | 1 | 0 | 0 | 0 | 1 | 1 | 1 | 1 |
| 159 | 0 | 0 | 0 | 1 | 1 | 1 | 1 | 1 | 1 | 1 |
| 160 | 1 | 1 | 1 | 0 | 0 | 0 | 0 | 0 | 0 | 0 |
| 161 | 1 | 1 | 1 | 1 | 1 | 1 | 1 | 1 | 1 | 1 |
| 162 | 1 | 1 | 1 | 1 | 1 | 1 | 1 | 1 | 1 | 1 |
| 163 | 1 | 1 | 1 | 1 | 1 | 1 | 1 | 1 | 1 | 1 |
| 164 | 1 | 1 | 1 | 1 | 1 | 1 | 1 | 1 | 1 | 1 |
| 165 | 1 | 1 | 1 | 1 | 1 | 1 | 1 | 1 | 1 | 1 |
| 166 | 1 | 1 | 1 | 1 | 1 | 1 | 1 | 1 | 1 | 1 |
| 167 | 1 | 1 | 1 | 1 | 1 | 1 | 1 | 1 | 1 | 1 |
| 168 | 1 | 1 | 1 | 0 | 0 | 0 | 0 | 0 | 0 | 0 |
| 169 | 1 | 1 | 0 | 0 | 0 | 0 | 0 | 0 | 0 | 0 |
| 170 | 1 | 0 | 0 | 1 | 1 | 1 | 1 | 1 | 1 | 1 |
| 171 | 0 | 1 | 0 | 0 | 0 | 0 | 0 | 0 | 0 | 0 |
| 172 | 0 | 0 | 0 | 0 | 0 | 0 | 0 | 0 | 0 | 0 |
| 173 | 1 | 1 | 1 | 1 | 1 | 1 | 1 | 1 | 1 | 1 |
| 174 | 0 | 0 | 0 | 0 | 0 | 0 | 0 | 0 | 0 | 0 |
| 175 | 1 | 1 | 1 | 1 | 1 | 1 | 1 | 1 | 1 | 1 |
| 176 | 1 | 0 | 0 | 1 | 0 | 0 | 0 | 0 | 1 | 0 |
| 177 | 0 | 0 | 0 | 0 | 0 | 0 | 0 | 0 | 0 | 0 |
| 178 | 0 | 0 | 0 | 0 | 0 | 0 | 1 | 0 | 0 | 1 |
| 179 | 0 | 0 | 0 | 0 | 0 | 0 | 0 | 0 | 0 | 0 |
| 180 | 0 | 1 | 0 | 0 | 1 | 1 | 0 | 0 | 1 | 1 |
| 181 | 1 | 1 | 1 | 1 | 1 | 1 | 1 | 1 | 1 | 1 |
| 182 | 1 | 1 | 1 | 1 | 1 | 1 | 1 | 1 | 1 | 1 |
| 183 | 1 | 1 | 1 | 1 | 1 | 1 | 1 | 1 | 1 | 1 |
| 184 | 1 | 1 | 1 | 0 | 1 | 0 | 0 | 0 | 1 | 0 |
| 185 | 0 | 1 | 0 | 0 | 0 | 0 | 0 | 0 | 0 | 0 |
| 186 | 0 | 0 | 0 | 1 | 0 | 0 | 1 | 0 | 0 | 0 |
| 187 | 0 | 0 | 0 | 0 | 0 | 0 | 0 | 0 | 0 | 0 |
| 188 | 1 | 0 | 0 | 0 | 0 | 0 | 0 | 0 | 0 | 0 |
| 189 | 0 | 1 | 0 | 1 | 0 | 1 | 0 | 0 | 1 | 0 |
| 190 | 0 | 0 | 0 | 0 | 0 | 0 | 0 | 0 | 0 | 0 |
| 191 | 1 | 1 | 1 | 1 | 1 | 1 | 1 | 1 | 1 | 1 |
| 192 | 0 | 0 | 0 | 0 | 0 | 0 | 0 | 0 | 0 | 0 |
| 193 | 1 | 1 | 0 | 0 | 1 | 1 | 1 | 1 | 1 | 1 |
| 194 | 1 | 1 | 1 | 1 | 1 | 1 | 1 | 1 | 1 | 1 |
| 195 | 1 | 1 | 1 | 1 | 1 | 1 | 1 | 1 | 1 | 1 |
| 196 | 0 | 0 | 0 | 0 | 0 | 0 | 0 | 0 | 0 | 0 |
| 197 | 1 | 1 | 1 | 1 | 1 | 1 | 1 | 1 | 1 | 1 |
| 198 | 0 | 0 | 0 | 0 | 0 | 0 | 0 | 0 | 0 | 0 |
| 199 | 1 | 1 | 1 | 1 | 1 | 1 | 1 | 1 | 1 | 1 |
| 200 | 0 | 0 | 0 | 0 | 0 | 0 | 0 | 0 | 0 | 0 |

| **ID** | **GPT_3.5_1** | **GPT_3.5_2** | **GPT_3.5_3** | **GPT_3.5_4** | **GPT_3.5_5** | **GPT_3.5_6** | **GPT_3.5_7** | **GPT_3.5_8** | **GPT_3.5_9** | **GPT_3.5_10** |
| --- | --- | --- | --- | --- | --- | --- | --- | --- | --- | --- |
| **1** | **0** | **0** | **0** | **0** | **0** | **0** | **0** | **0** | **0** | **0** |
| **2** | **0** | **0** | **0** | **0** | **0** | **0** | **0** | **0** | **0** | **0** |
| **3** | **0** | **0** | **0** | **0** | **0** | **0** | **0** | **0** | **0** | **0** |
| **4** | **0** | **0** | **0** | **0** | **0** | **0** | **0** | **0** | **0** | **0** |
| **5** | **1** | **1** | **1** | **1** | **1** | **1** | **1** | **1** | **1** | **1** |
| **6** | **1** | **1** | **1** | **1** | **1** | **1** | **1** | **1** | **1** | **1** |
| **7** | **0** | **0** | **0** | **0** | **0** | **0** | **0** | **0** | **0** | **0** |
| **8** | **1** | **1** | **1** | **1** | **1** | **1** | **1** | **1** | **1** | **1** |
| **9** | **0** | **1** | **0** | **1** | **1** | **1** | **0** | **1** | **0** | **0** |
| **10** | **1** | **1** | **1** | **1** | **1** | **1** | **1** | **1** | **1** | **1** |
| **11** | **0** | **0** | **0** | **0** | **0** | **0** | **0** | **0** | **0** | **0** |
| **12** | **1** | **1** | **0** | **0** | **0** | **1** | **0** | **0** | **1** | **1** |
| **13** | **0** | **0** | **1** | **0** | **0** | **0** | **0** | **0** | **0** | **0** |
| **14** | **0** | **0** | **0** | **0** | **0** | **0** | **1** | **0** | **0** | **0** |
| **15** | **1** | **1** | **1** | **1** | **1** | **1** | **1** | **1** | **1** | **1** |
| **16** | **1** | **0** | **1** | **1** | **0** | **1** | **1** | **1** | **0** | **0** |
| **17** | **1** | **1** | **1** | **1** | **1** | **1** | **1** | **1** | **1** | **1** |
| **18** | **0** | **0** | **0** | **0** | **0** | **0** | **0** | **0** | **0** | **0** |
| **19** | **0** | **0** | **0** | **0** | **0** | **0** | **0** | **0** | **0** | **0** |
| **20** | **0** | **0** | **0** | **0** | **0** | **0** | **0** | **0** | **0** | **0** |
| **21** | **1** | **1** | **1** | **1** | **1** | **1** | **1** | **1** | **1** | **1** |
| **22** | **0** | **0** | **0** | **0** | **0** | **0** | **0** | **0** | **0** | **0** |
| **23** | **1** | **1** | **1** | **1** | **1** | **1** | **1** | **1** | **1** | **1** |
| **24** | **0** | **0** | **0** | **0** | **0** | **0** | **0** | **0** | **0** | **0** |
| **25** | **0** | **1** | **1** | **1** | **1** | **1** | **1** | **1** | **1** | **1** |
| **26** | **0** | **0** | **0** | **0** | **0** | **0** | **0** | **1** | **0** | **0** |
| **27** | **0** | **0** | **0** | **0** | **0** | **0** | **0** | **0** | **0** | **0** |
| **28** | **1** | **1** | **1** | **1** | **1** | **1** | **1** | **1** | **1** | **1** |
| **29** | **0** | **0** | **0** | **0** | **0** | **0** | **0** | **0** | **0** | **0** |
| **30** | **1** | **1** | **1** | **1** | **1** | **1** | **1** | **1** | **1** | **1** |
| **31** | **1** | **1** | **1** | **1** | **1** | **1** | **1** | **1** | **1** | **1** |
| **32** | **0** | **0** | **0** | **0** | **0** | **0** | **0** | **1** | **0** | **0** |
| **33** | **1** | **0** | **1** | **1** | **0** | **1** | **0** | **1** | **0** | **0** |
| **34** | **1** | **1** | **1** | **1** | **1** | **1** | **1** | **1** | **1** | **1** |
| **35** | **0** | **0** | **1** | **0** | **0** | **1** | **0** | **1** | **1** | **0** |
| **36** | **1** | **1** | **0** | **1** | **1** | **1** | **0** | **0** | **1** | **1** |
| **37** | **0** | **0** | **0** | **0** | **0** | **0** | **0** | **0** | **0** | **0** |
| **38** | **0** | **0** | **0** | **0** | **0** | **0** | **0** | **0** | **0** | **0** |
| **39** | **0** | **0** | **0** | **0** | **0** | **0** | **0** | **0** | **0** | **0** |
| **40** | **1** | **1** | **1** | **1** | **1** | **1** | **1** | **1** | **1** | **1** |
| **41** | **0** | **0** | **0** | **0** | **0** | **0** | **0** | **0** | **0** | **0** |
| **42** | **0** | **0** | **0** | **0** | **0** | **0** | **0** | **0** | **0** | **0** |
| **43** | **0** | **0** | **0** | **0** | **0** | **0** | **0** | **0** | **0** | **0** |
| **44** | **1** | **1** | **1** | **1** | **1** | **1** | **1** | **1** | **1** | **1** |
| **45** | **1** | **1** | **1** | **1** | **1** | **1** | **1** | **1** | **1** | **1** |
| **46** | **1** | **1** | **1** | **1** | **1** | **1** | **1** | **1** | **1** | **1** |
| **47** | **0** | **0** | **0** | **0** | **0** | **0** | **0** | **0** | **0** | **0** |
| **48** | **0** | **0** | **0** | **0** | **0** | **0** | **0** | **0** | **0** | **0** |
| **49** | **1** | **1** | **1** | **1** | **1** | **1** | **1** | **1** | **1** | **1** |
| **50** | **0** | **0** | **0** | **0** | **0** | **0** | **0** | **0** | **0** | **0** |
| **51** | **0** | **0** | **0** | **0** | **0** | **0** | **0** | **0** | **0** | **0** |
| **52** | **0** | **0** | **0** | **0** | **0** | **0** | **0** | **0** | **0** | **0** |
| **53** | **0** | **0** | **0** | **0** | **0** | **0** | **0** | **0** | **0** | **0** |
| **54** | **0** | **0** | **0** | **0** | **0** | **0** | **0** | **0** | **0** | **0** |
| **55** | **1** | **1** | **1** | **1** | **1** | **1** | **1** | **1** | **1** | **1** |
| **56** | **1** | **1** | **1** | **1** | **1** | **1** | **1** | **1** | **1** | **1** |
| **57** | **0** | **0** | **0** | **0** | **0** | **0** | **0** | **0** | **0** | **0** |
| **58** | **1** | **1** | **1** | **1** | **1** | **1** | **1** | **1** | **1** | **1** |
| **59** | **1** | **1** | **1** | **1** | **1** | **1** | **1** | **1** | **1** | **1** |
| **60** | **0** | **0** | **0** | **0** | **0** | **0** | **0** | **0** | **0** | **0** |
| **61** | **1** | **1** | **0** | **1** | **0** | **1** | **0** | **1** | **1** | **0** |
| **62** | **1** | **1** | **1** | **1** | **1** | **1** | **1** | **0** | **1** | **1** |
| **63** | **1** | **1** | **1** | **1** | **1** | **1** | **1** | **1** | **1** | **1** |
| **64** | **1** | **1** | **1** | **1** | **1** | **1** | **1** | **1** | **1** | **1** |
| **65** | **0** | **0** | **0** | **0** | **0** | **0** | **0** | **0** | **0** | **0** |
| **66** | **1** | **1** | **1** | **1** | **1** | **1** | **1** | **1** | **1** | **1** |
| **67** | **1** | **1** | **1** | **1** | **1** | **1** | **1** | **1** | **1** | **1** |
| **68** | **1** | **1** | **1** | **1** | **1** | **1** | **1** | **1** | **1** | **1** |
| **69** | **1** | **1** | **1** | **1** | **1** | **1** | **1** | **1** | **1** | **1** |
| **70** | **0** | **0** | **0** | **0** | **0** | **0** | **0** | **0** | **0** | **0** |
| **71** | **0** | **0** | **0** | **0** | **0** | **0** | **0** | **0** | **0** | **0** |
| **72** | **1** | **1** | **1** | **1** | **1** | **1** | **1** | **1** | **1** | **1** |
| **73** | **1** | **1** | **1** | **1** | **1** | **1** | **1** | **1** | **1** | **1** |
| **74** | **1** | **1** | **1** | **1** | **1** | **1** | **1** | **1** | **1** | **1** |
| **75** | **1** | **1** | **1** | **1** | **1** | **1** | **1** | **1** | **1** | **1** |
| **76** | **1** | **1** | **1** | **1** | **1** | **1** | **1** | **1** | **1** | **1** |
| **77** | **0** | **0** | **0** | **0** | **0** | **0** | **0** | **0** | **0** | **0** |
| **78** | **0** | **1** | **0** | **0** | **1** | **1** | **1** | **0** | **0** | **0** |
| **79** | **1** | **1** | **1** | **1** | **1** | **1** | **1** | **1** | **1** | **1** |
| **80** | **0** | **0** | **0** | **0** | **0** | **0** | **0** | **0** | **0** | **0** |
| **81** | **0** | **0** | **0** | **0** | **0** | **0** | **0** | **0** | **0** | **0** |
| **82** | **0** | **0** | **0** | **0** | **0** | **0** | **0** | **0** | **0** | **0** |
| **83** | **1** | **1** | **1** | **1** | **1** | **1** | **1** | **1** | **1** | **1** |
| **84** | **0** | **0** | **0** | **0** | **0** | **0** | **0** | **0** | **0** | **0** |
| **85** | **0** | **0** | **0** | **0** | **0** | **0** | **0** | **0** | **0** | **0** |
| **86** | **0** | **0** | **0** | **0** | **0** | **0** | **0** | **0** | **0** | **0** |
| **87** | **0** | **0** | **0** | **0** | **0** | **0** | **0** | **0** | **0** | **0** |
| **88** | **1** | **1** | **1** | **1** | **1** | **1** | **1** | **1** | **1** | **1** |
| **89** | **0** | **0** | **0** | **0** | **0** | **0** | **0** | **0** | **0** | **0** |
| **90** | **1** | **1** | **1** | **1** | **1** | **1** | **1** | **1** | **1** | **1** |
| **91** | **1** | **1** | **1** | **1** | **1** | **1** | **1** | **1** | **1** | **1** |
| **92** | **1** | **1** | **1** | **1** | **1** | **1** | **1** | **1** | **1** | **1** |
| **93** | **1** | **1** | **1** | **1** | **1** | **1** | **1** | **1** | **1** | **1** |
| **94** | **0** | **0** | **0** | **0** | **0** | **0** | **0** | **0** | **0** | **0** |
| **95** | **1** | **1** | **0** | **1** | **0** | **1** | **1** | **1** | **1** | **0** |
| **96** | **1** | **1** | **1** | **1** | **1** | **1** | **1** | **1** | **1** | **1** |
| **97** | **1** | **1** | **1** | **1** | **1** | **1** | **1** | **1** | **1** | **1** |
| **98** | **0** | **0** | **0** | **0** | **1** | **1** | **1** | **1** | **1** | **1** |
| **99** | **1** | **0** | **0** | **0** | **0** | **0** | **0** | **0** | **0** | **0** |
| **100** | **1** | **1** | **1** | **1** | **1** | **1** | **1** | **1** | **1** | **1** |
| **101** | **1** | **0** | **1** | **0** | **1** | **1** | **1** | **1** | **1** | **1** |
| **102** | **1** | **1** | **1** | **1** | **1** | **1** | **1** | **1** | **1** | **1** |
| **103** | **0** | **0** | **0** | **0** | **0** | **0** | **0** | **0** | **0** | **0** |
| **104** | **0** | **1** | **1** | **1** | **1** | **1** | **1** | **1** | **1** | **1** |
| **105** | **1** | **1** | **1** | **1** | **1** | **1** | **1** | **1** | **1** | **1** |
| **106** | **0** | **0** | **0** | **0** | **0** | **0** | **0** | **0** | **0** | **0** |
| **107** | **1** | **1** | **1** | **1** | **1** | **1** | **1** | **1** | **1** | **1** |
| **108** | **0** | **0** | **0** | **0** | **0** | **0** | **0** | **0** | **0** | **0** |
| **109** | **1** | **1** | **1** | **1** | **1** | **1** | **1** | **1** | **1** | **1** |
| **110** | **0** | **0** | **0** | **0** | **0** | **0** | **0** | **0** | **0** | **0** |
| **111** | **1** | **1** | **1** | **1** | **1** | **1** | **1** | **1** | **1** | **1** |
| **112** | **0** | **0** | **0** | **0** | **0** | **0** | **0** | **0** | **0** | **0** |
| **113** | **1** | **1** | **1** | **1** | **1** | **1** | **1** | **1** | **1** | **1** |
| **114** | **1** | **1** | **1** | **1** | **1** | **1** | **1** | **1** | **1** | **1** |
| **115** | **0** | **0** | **0** | **0** | **0** | **0** | **0** | **0** | **0** | **0** |
| **116** | **1** | **0** | **0** | **1** | **1** | **0** | **1** | **1** | **1** | **1** |
| **117** | **0** | **0** | **0** | **0** | **0** | **0** | **0** | **0** | **0** | **0** |
| **118** | **1** | **1** | **1** | **1** | **1** | **1** | **1** | **1** | **1** | **1** |
| **119** | **1** | **1** | **1** | **1** | **1** | **1** | **1** | **1** | **1** | **1** |
| **120** | **1** | **1** | **1** | **1** | **1** | **1** | **1** | **1** | **1** | **1** |
| **121** | **1** | **0** | **1** | **0** | **0** | **0** | **0** | **1** | **0** | **0** |
| **122** | **0** | **0** | **0** | **0** | **0** | **0** | **0** | **0** | **0** | **0** |
| **123** | **1** | **1** | **1** | **1** | **1** | **1** | **1** | **1** | **1** | **1** |
| **124** | **1** | **1** | **1** | **1** | **1** | **1** | **1** | **1** | **1** | **1** |
| **125** | **1** | **1** | **1** | **1** | **1** | **1** | **1** | **1** | **1** | **1** |
| **126** | **0** | **0** | **0** | **0** | **0** | **0** | **0** | **0** | **0** | **0** |
| **127** | **0** | **0** | **0** | **0** | **0** | **0** | **0** | **0** | **0** | **0** |
| **128** | **1** | **1** | **1** | **1** | **1** | **1** | **1** | **1** | **1** | **1** |
| **129** | **0** | **0** | **0** | **0** | **0** | **0** | **0** | **0** | **0** | **0** |
| **130** | **1** | **1** | **1** | **1** | **1** | **1** | **1** | **1** | **1** | **1** |
| **131** | **0** | **0** | **0** | **0** | **0** | **0** | **0** | **0** | **0** | **0** |
| **132** | **0** | **0** | **0** | **0** | **0** | **0** | **0** | **0** | **0** | **0** |
| **133** | **1** | **1** | **1** | **1** | **1** | **1** | **1** | **1** | **1** | **1** |
| **134** | **1** | **1** | **1** | **1** | **1** | **1** | **1** | **1** | **1** | **1** |
| **135** | **0** | **0** | **0** | **0** | **0** | **0** | **0** | **0** | **0** | **0** |
| **136** | **1** | **1** | **1** | **1** | **1** | **1** | **1** | **1** | **1** | **1** |
| **137** | **0** | **0** | **0** | **0** | **0** | **0** | **0** | **0** | **0** | **0** |
| **138** | **0** | **0** | **0** | **0** | **0** | **0** | **0** | **0** | **0** | **0** |
| **139** | **0** | **0** | **0** | **0** | **0** | **0** | **0** | **0** | **0** | **0** |
| **140** | **1** | **1** | **1** | **1** | **1** | **1** | **1** | **1** | **1** | **1** |
| **141** | **0** | **0** | **0** | **1** | **1** | **0** | **0** | **0** | **0** | **1** |
| **142** | **1** | **1** | **1** | **1** | **1** | **1** | **1** | **1** | **1** | **1** |
| **143** | **0** | **0** | **1** | **1** | **0** | **1** | **1** | **0** | **0** | **0** |
| **144** | **1** | **1** | **1** | **0** | **1** | **0** | **0** | **0** | **1** | **0** |
| **145** | **0** | **0** | **0** | **0** | **0** | **0** | **0** | **0** | **0** | **0** |
| **146** | **0** | **0** | **0** | **0** | **0** | **0** | **0** | **0** | **0** | **0** |
| **147** | **1** | **1** | **1** | **1** | **1** | **1** | **1** | **1** | **1** | **1** |
| **148** | **0** | **0** | **0** | **0** | **0** | **0** | **0** | **0** | **0** | **0** |
| **149** | **0** | **0** | **0** | **0** | **0** | **0** | **0** | **0** | **0** | **0** |
| **150** | **0** | **0** | **0** | **0** | **0** | **0** | **0** | **0** | **0** | **0** |
| **151** | **1** | **1** | **1** | **1** | **1** | **1** | **1** | **1** | **1** | **1** |
| **152** | **0** | **0** | **1** | **0** | **0** | **0** | **0** | **0** | **0** | **0** |
| **153** | **0** | **0** | **0** | **0** | **0** | **0** | **0** | **0** | **0** | **0** |
| **154** | **0** | **0** | **0** | **0** | **0** | **0** | **0** | **0** | **0** | **0** |
| **155** | **1** | **1** | **1** | **1** | **1** | **1** | **1** | **1** | **1** | **1** |
| **156** | **1** | **0** | **0** | **0** | **0** | **0** | **1** | **0** | **0** | **0** |
| **157** | **0** | **0** | **0** | **0** | **0** | **0** | **0** | **0** | **0** | **0** |
| **158** | **0** | **0** | **0** | **1** | **1** | **0** | **1** | **0** | **0** | **0** |
| **159** | **1** | **1** | **1** | **1** | **1** | **1** | **1** | **1** | **1** | **1** |
| **160** | **1** | **1** | **1** | **0** | **0** | **0** | **0** | **1** | **0** | **0** |
| **161** | **1** | **1** | **1** | **1** | **1** | **1** | **1** | **1** | **1** | **1** |
| **162** | **1** | **1** | **1** | **1** | **1** | **1** | **1** | **1** | **1** | **1** |
| **163** | **1** | **1** | **1** | **1** | **1** | **1** | **1** | **1** | **1** | **1** |
| **164** | **1** | **1** | **1** | **1** | **1** | **1** | **1** | **1** | **1** | **1** |
| **165** | **0** | **1** | **0** | **1** | **1** | **0** | **1** | **1** | **1** | **0** |
| **166** | **1** | **1** | **1** | **1** | **1** | **1** | **1** | **1** | **1** | **1** |
| **167** | **1** | **1** | **1** | **1** | **1** | **1** | **1** | **1** | **1** | **1** |
| **168** | **0** | **0** | **0** | **0** | **0** | **0** | **0** | **0** | **0** | **0** |
| **169** | **0** | **0** | **0** | **0** | **0** | **0** | **0** | **0** | **0** | **0** |
| **170** | **1** | **1** | **1** | **1** | **1** | **1** | **1** | **1** | **1** | **1** |
| **171** | **0** | **0** | **0** | **0** | **0** | **0** | **0** | **0** | **0** | **0** |
| **172** | **0** | **0** | **0** | **0** | **0** | **0** | **0** | **0** | **0** | **0** |
| **173** | **1** | **1** | **1** | **1** | **1** | **1** | **1** | **1** | **1** | **1** |
| **174** | **0** | **0** | **0** | **0** | **0** | **0** | **0** | **0** | **0** | **0** |
| **175** | **1** | **1** | **1** | **1** | **1** | **1** | **1** | **1** | **1** | **1** |
| **176** | **1** | **1** | **1** | **1** | **1** | **1** | **1** | **1** | **0** | **1** |
| **177** | **0** | **0** | **0** | **0** | **0** | **0** | **0** | **0** | **0** | **0** |
| **178** | **0** | **0** | **0** | **0** | **0** | **0** | **0** | **0** | **0** | **0** |
| **179** | **0** | **0** | **0** | **0** | **0** | **0** | **0** | **0** | **0** | **0** |
| **180** | **1** | **1** | **1** | **1** | **1** | **1** | **1** | **1** | **1** | **1** |
| **181** | **1** | **0** | **1** | **1** | **1** | **1** | **1** | **1** | **1** | **1** |
| **182** | **1** | **1** | **1** | **1** | **1** | **1** | **1** | **1** | **1** | **1** |
| **183** | **1** | **1** | **1** | **1** | **1** | **1** | **1** | **1** | **1** | **1** |
| **184** | **0** | **0** | **0** | **0** | **0** | **0** | **0** | **0** | **0** | **0** |
| **185** | **0** | **0** | **0** | **0** | **0** | **0** | **0** | **0** | **0** | **0** |
| **186** | **0** | **0** | **0** | **0** | **0** | **0** | **0** | **0** | **0** | **0** |
| **187** | **0** | **0** | **0** | **0** | **0** | **0** | **0** | **0** | **0** | **0** |
| **188** | **0** | **0** | **0** | **0** | **0** | **0** | **0** | **0** | **0** | **0** |
| **189** | **0** | **0** | **0** | **0** | **0** | **0** | **0** | **0** | **0** | **0** |
| **190** | **0** | **0** | **0** | **0** | **0** | **0** | **0** | **0** | **0** | **0** |
| **191** | **1** | **1** | **1** | **1** | **1** | **1** | **1** | **1** | **1** | **1** |
| **192** | **0** | **0** | **0** | **0** | **0** | **0** | **0** | **0** | **0** | **0** |
| **193** | **0** | **0** | **0** | **0** | **0** | **0** | **0** | **0** | **0** | **0** |
| **194** | **1** | **1** | **1** | **1** | **1** | **1** | **1** | **1** | **1** | **1** |
| **195** | **1** | **1** | **1** | **1** | **1** | **1** | **1** | **1** | **1** | **1** |
| **196** | **0** | **0** | **1** | **0** | **0** | **1** | **0** | **1** | **0** | **0** |
| **197** | **1** | **1** | **1** | **1** | **1** | **1** | **1** | **1** | **1** | **1** |
| **198** | **0** | **0** | **0** | **0** | **0** | **0** | **0** | **0** | **0** | **0** |
| **199** | **1** | **1** | **1** | **1** | **1** | **1** | **1** | **1** | **1** | **1** |
| **200** | **0** | **0** | **0** | **0** | **0** | **0** | **0** | **0** | **0** | **0** |

| **ID** | **GPT_4_1** | **GPT_4_2** | **GPT_4_3** | **GPT_4_4** | **GPT_4_5** | **GPT_4_6** | **GPT_4_7** | **GPT_4_8** | **GPT_4_9** | **GPT_4_10** |
| --- | --- | --- | --- | --- | --- | --- | --- | --- | --- | --- |
| **1** | **1** | **1** | **1** | **1** | **1** | **1** | **1** | **1** | **1** | **1** |
| **2** | **0** | **1** | **0** | **1** | **0** | **1** | **1** | **0** | **1** | **1** |
| **3** | **1** | **1** | **1** | **1** | **1** | **1** | **1** | **1** | **1** | **1** |
| **4** | **1** | **1** | **1** | **1** | **1** | **1** | **1** | **1** | **1** | **1** |
| **5** | **1** | **1** | **1** | **1** | **1** | **1** | **1** | **1** | **1** | **1** |
| **6** | **1** | **1** | **1** | **1** | **1** | **1** | **1** | **1** | **1** | **1** |
| **7** | **1** | **1** | **1** | **1** | **1** | **1** | **1** | **1** | **1** | **1** |
| **8** | **0** | **1** | **0** | **0** | **0** | **0** | **0** | **1** | **0** | **0** |
| **9** | **1** | **1** | **1** | **1** | **1** | **1** | **1** | **1** | **1** | **1** |
| **10** | **1** | **1** | **1** | **1** | **1** | **1** | **1** | **1** | **1** | **1** |
| **11** | **0** | **0** | **0** | **0** | **0** | **0** | **0** | **0** | **0** | **0** |
| **12** | **0** | **0** | **0** | **0** | **0** | **0** | **0** | **0** | **0** | **0** |
| **13** | **0** | **0** | **0** | **0** | **0** | **0** | **0** | **0** | **0** | **0** |
| **14** | **1** | **1** | **1** | **1** | **1** | **1** | **1** | **1** | **1** | **1** |
| **15** | **1** | **1** | **1** | **1** | **1** | **1** | **1** | **1** | **1** | **1** |
| **16** | **1** | **1** | **0** | **1** | **1** | **0** | **0** | **1** | **1** | **0** |
| **17** | **1** | **1** | **1** | **1** | **1** | **1** | **1** | **1** | **1** | **1** |
| **18** | **1** | **1** | **1** | **1** | **1** | **1** | **1** | **1** | **1** | **1** |
| **19** | **0** | **0** | **0** | **0** | **0** | **0** | **0** | **0** | **0** | **0** |
| **20** | **0** | **0** | **0** | **1** | **0** | **0** | **1** | **0** | **0** | **0** |
| **21** | **1** | **1** | **1** | **1** | **1** | **1** | **1** | **1** | **1** | **1** |
| **22** | **0** | **0** | **0** | **0** | **0** | **0** | **0** | **0** | **0** | **0** |
| **23** | **1** | **1** | **1** | **1** | **1** | **1** | **1** | **1** | **1** | **1** |
| **24** | **0** | **0** | **0** | **0** | **0** | **0** | **0** | **0** | **0** | **0** |
| **25** | **1** | **1** | **1** | **1** | **1** | **1** | **1** | **1** | **1** | **1** |
| **26** | **0** | **0** | **0** | **0** | **0** | **0** | **0** | **0** | **0** | **0** |
| **27** | **0** | **0** | **0** | **0** | **0** | **0** | **0** | **0** | **1** | **0** |
| **28** | **1** | **1** | **1** | **1** | **1** | **1** | **1** | **1** | **1** | **1** |
| **29** | **0** | **0** | **0** | **0** | **0** | **0** | **0** | **0** | **0** | **0** |
| **30** | **1** | **1** | **1** | **1** | **1** | **1** | **1** | **1** | **1** | **1** |
| **31** | **1** | **0** | **1** | **1** | **1** | **0** | **1** | **1** | **0** | **0** |
| **32** | **1** | **1** | **1** | **1** | **1** | **1** | **1** | **1** | **1** | **1** |
| **33** | **1** | **1** | **1** | **1** | **1** | **1** | **1** | **1** | **1** | **1** |
| **34** | **1** | **1** | **1** | **1** | **1** | **1** | **1** | **1** | **1** | **1** |
| **35** | **1** | **1** | **1** | **1** | **1** | **1** | **1** | **1** | **1** | **1** |
| **36** | **1** | **1** | **1** | **1** | **1** | **1** | **1** | **1** | **1** | **1** |
| **37** | **0** | **0** | **0** | **0** | **0** | **0** | **0** | **0** | **0** | **0** |
| **38** | **0** | **0** | **0** | **0** | **0** | **0** | **0** | **0** | **0** | **0** |
| **39** | **0** | **0** | **0** | **0** | **0** | **0** | **0** | **0** | **0** | **0** |
| **40** | **1** | **1** | **1** | **1** | **1** | **1** | **1** | **1** | **1** | **1** |
| **41** | **1** | **1** | **1** | **1** | **1** | **1** | **1** | **1** | **1** | **1** |
| **42** | **0** | **0** | **0** | **0** | **0** | **0** | **0** | **0** | **0** | **0** |
| **43** | **0** | **0** | **0** | **0** | **0** | **0** | **0** | **0** | **0** | **0** |
| **44** | **1** | **1** | **1** | **1** | **1** | **1** | **1** | **1** | **1** | **1** |
| **45** | **1** | **1** | **1** | **1** | **1** | **1** | **1** | **1** | **1** | **1** |
| **46** | **1** | **1** | **1** | **1** | **1** | **1** | **1** | **1** | **1** | **1** |
| **47** | **1** | **0** | **1** | **0** | **1** | **0** | **0** | **0** | **0** | **1** |
| **48** | **1** | **0** | **1** | **0** | **1** | **0** | **0** | **1** | **0** | **1** |
| **49** | **1** | **1** | **1** | **1** | **1** | **1** | **1** | **1** | **1** | **1** |
| **50** | **0** | **1** | **1** | **1** | **0** | **0** | **1** | **1** | **1** | **0** |
| **51** | **0** | **0** | **0** | **0** | **0** | **0** | **0** | **0** | **0** | **0** |
| **52** | **1** | **1** | **1** | **1** | **1** | **1** | **1** | **1** | **1** | **1** |
| **53** | **1** | **1** | **1** | **1** | **1** | **1** | **1** | **1** | **1** | **1** |
| **54** | **1** | **1** | **1** | **1** | **1** | **1** | **1** | **1** | **1** | **1** |
| **55** | **1** | **1** | **1** | **1** | **1** | **1** | **1** | **1** | **1** | **1** |
| **56** | **1** | **1** | **1** | **1** | **1** | **1** | **1** | **1** | **1** | **1** |
| **57** | **1** | **1** | **1** | **1** | **1** | **1** | **1** | **1** | **1** | **1** |
| **58** | **1** | **1** | **1** | **1** | **1** | **1** | **1** | **1** | **1** | **1** |
| **59** | **1** | **1** | **1** | **1** | **1** | **1** | **1** | **1** | **1** | **1** |
| **60** | **1** | **1** | **1** | **1** | **1** | **1** | **1** | **1** | **1** | **1** |
| **61** | **1** | **1** | **1** | **1** | **1** | **1** | **1** | **1** | **1** | **1** |
| **62** | **0** | **0** | **0** | **0** | **0** | **0** | **0** | **0** | **0** | **0** |
| **63** | **1** | **1** | **1** | **1** | **1** | **1** | **1** | **1** | **1** | **1** |
| **64** | **1** | **1** | **1** | **1** | **1** | **1** | **1** | **1** | **1** | **1** |
| **65** | **1** | **1** | **1** | **1** | **1** | **1** | **1** | **1** | **1** | **1** |
| **66** | **1** | **1** | **1** | **1** | **1** | **1** | **1** | **1** | **1** | **1** |
| **67** | **1** | **1** | **1** | **1** | **1** | **1** | **1** | **1** | **1** | **1** |
| **68** | **0** | **0** | **0** | **0** | **0** | **1** | **0** | **0** | **0** | **0** |
| **69** | **1** | **1** | **1** | **1** | **1** | **1** | **1** | **1** | **1** | **1** |
| **70** | **1** | **1** | **1** | **1** | **1** | **1** | **1** | **1** | **1** | **1** |
| **71** | **1** | **1** | **1** | **1** | **1** | **1** | **1** | **1** | **1** | **1** |
| **72** | **1** | **1** | **1** | **1** | **1** | **1** | **1** | **1** | **1** | **1** |
| **73** | **1** | **1** | **1** | **1** | **1** | **1** | **1** | **1** | **1** | **1** |
| **74** | **1** | **1** | **1** | **1** | **1** | **1** | **1** | **1** | **1** | **1** |
| **75** | **1** | **1** | **1** | **1** | **1** | **1** | **1** | **1** | **1** | **1** |
| **76** | **1** | **1** | **1** | **1** | **1** | **1** | **1** | **1** | **1** | **1** |
| **77** | **1** | **1** | **1** | **1** | **1** | **1** | **1** | **1** | **1** | **1** |
| **78** | **1** | **1** | **1** | **1** | **1** | **1** | **1** | **1** | **1** | **1** |
| **79** | **1** | **1** | **1** | **1** | **1** | **1** | **1** | **1** | **1** | **1** |
| **80** | **0** | **0** | **0** | **0** | **0** | **0** | **0** | **0** | **0** | **0** |
| **81** | **0** | **0** | **0** | **0** | **0** | **0** | **0** | **0** | **0** | **0** |
| **82** | **1** | **1** | **1** | **1** | **1** | **1** | **1** | **1** | **1** | **1** |
| **83** | **1** | **1** | **1** | **1** | **1** | **1** | **1** | **1** | **1** | **1** |
| **84** | **1** | **1** | **1** | **1** | **1** | **1** | **1** | **1** | **1** | **1** |
| **85** | **0** | **0** | **0** | **0** | **0** | **0** | **0** | **0** | **0** | **0** |
| **86** | **0** | **0** | **0** | **0** | **0** | **0** | **0** | **0** | **0** | **0** |
| **87** | **1** | **1** | **1** | **1** | **1** | **1** | **1** | **1** | **1** | **1** |
| **88** | **0** | **0** | **0** | **0** | **0** | **0** | **0** | **0** | **0** | **0** |
| **89** | **1** | **1** | **1** | **1** | **1** | **1** | **1** | **1** | **1** | **1** |
| **90** | **1** | **1** | **1** | **1** | **1** | **1** | **1** | **1** | **1** | **1** |
| **91** | **1** | **1** | **1** | **1** | **1** | **1** | **1** | **1** | **1** | **1** |
| **92** | **1** | **1** | **1** | **1** | **1** | **1** | **1** | **1** | **1** | **1** |
| **93** | **1** | **1** | **1** | **1** | **1** | **1** | **1** | **1** | **1** | **1** |
| **94** | **1** | **1** | **1** | **1** | **1** | **1** | **1** | **1** | **1** | **1** |
| **95** | **1** | **1** | **1** | **1** | **1** | **1** | **1** | **1** | **1** | **1** |
| **96** | **1** | **1** | **1** | **1** | **1** | **1** | **1** | **1** | **1** | **1** |
| **97** | **1** | **1** | **1** | **1** | **1** | **1** | **1** | **1** | **1** | **1** |
| **98** | **0** | **0** | **0** | **0** | **0** | **0** | **0** | **0** | **0** | **0** |
| **99** | **1** | **1** | **1** | **1** | **1** | **1** | **1** | **1** | **0** | **1** |
| **100** | **1** | **1** | **1** | **1** | **1** | **1** | **1** | **1** | **1** | **1** |
| **101** | **1** | **1** | **1** | **1** | **1** | **1** | **1** | **1** | **1** | **1** |
| **102** | **1** | **1** | **1** | **1** | **1** | **1** | **1** | **1** | **1** | **1** |
| **103** | **1** | **1** | **1** | **1** | **1** | **1** | **1** | **1** | **1** | **1** |
| **104** | **0** | **0** | **1** | **0** | **1** | **0** | **0** | **0** | **0** | **0** |
| **105** | **1** | **1** | **1** | **1** | **1** | **1** | **1** | **1** | **1** | **1** |
| **106** | **0** | **0** | **0** | **0** | **0** | **0** | **0** | **0** | **0** | **0** |
| **107** | **1** | **1** | **1** | **1** | **1** | **1** | **1** | **1** | **1** | **1** |
| **108** | **1** | **1** | **1** | **1** | **1** | **1** | **1** | **1** | **1** | **1** |
| **109** | **0** | **0** | **0** | **0** | **0** | **0** | **0** | **0** | **0** | **0** |
| **110** | **0** | **0** | **0** | **0** | **0** | **0** | **0** | **0** | **0** | **0** |
| **111** | **1** | **1** | **1** | **1** | **1** | **1** | **1** | **1** | **1** | **1** |
| **112** | **1** | **1** | **1** | **1** | **1** | **1** | **1** | **1** | **1** | **1** |
| **113** | **1** | **1** | **1** | **1** | **1** | **1** | **1** | **1** | **1** | **1** |
| **114** | **1** | **1** | **1** | **1** | **1** | **1** | **1** | **1** | **1** | **1** |
| **115** | **0** | **0** | **0** | **0** | **0** | **0** | **0** | **0** | **0** | **0** |
| **116** | **1** | **1** | **1** | **1** | **1** | **1** | **1** | **1** | **1** | **1** |
| **117** | **0** | **0** | **0** | **0** | **0** | **0** | **0** | **0** | **0** | **0** |
| **118** | **1** | **1** | **1** | **1** | **1** | **1** | **1** | **1** | **1** | **1** |
| **119** | **1** | **1** | **1** | **1** | **1** | **1** | **1** | **1** | **1** | **1** |
| **120** | **1** | **1** | **1** | **1** | **1** | **1** | **1** | **1** | **1** | **1** |
| **121** | **0** | **0** | **0** | **0** | **0** | **0** | **0** | **0** | **0** | **0** |
| **122** | **1** | **1** | **1** | **1** | **1** | **1** | **1** | **1** | **1** | **1** |
| **123** | **1** | **1** | **1** | **1** | **1** | **1** | **1** | **1** | **1** | **1** |
| **124** | **1** | **1** | **1** | **1** | **1** | **1** | **1** | **1** | **1** | **1** |
| **125** | **1** | **1** | **1** | **1** | **1** | **1** | **1** | **1** | **1** | **1** |
| **126** | **0** | **0** | **0** | **0** | **0** | **0** | **0** | **0** | **0** | **0** |
| **127** | **0** | **0** | **0** | **0** | **0** | **0** | **0** | **0** | **0** | **0** |
| **128** | **1** | **1** | **1** | **1** | **1** | **1** | **1** | **1** | **1** | **1** |
| **129** | **0** | **0** | **0** | **1** | **0** | **1** | **0** | **0** | **0** | **0** |
| **130** | **1** | **1** | **1** | **1** | **1** | **1** | **1** | **1** | **1** | **1** |
| **131** | **1** | **1** | **1** | **1** | **1** | **1** | **1** | **1** | **1** | **1** |
| **132** | **1** | **1** | **1** | **1** | **1** | **1** | **1** | **1** | **1** | **1** |
| **133** | **1** | **1** | **1** | **1** | **1** | **1** | **1** | **1** | **1** | **1** |
| **134** | **1** | **1** | **1** | **1** | **1** | **1** | **1** | **1** | **1** | **1** |
| **135** | **0** | **0** | **0** | **0** | **0** | **0** | **0** | **0** | **0** | **0** |
| **136** | **1** | **1** | **1** | **1** | **1** | **1** | **1** | **1** | **1** | **1** |
| **137** | **1** | **1** | **1** | **1** | **1** | **1** | **1** | **1** | **1** | **1** |
| **138** | **1** | **1** | **1** | **1** | **1** | **1** | **1** | **1** | **1** | **1** |
| **139** | **0** | **0** | **0** | **0** | **0** | **0** | **0** | **0** | **0** | **0** |
| **140** | **1** | **1** | **1** | **1** | **1** | **1** | **1** | **1** | **1** | **1** |
| **141** | **1** | **1** | **1** | **1** | **1** | **1** | **1** | **1** | **1** | **1** |
| **142** | **1** | **1** | **1** | **1** | **1** | **1** | **1** | **1** | **1** | **1** |
| **143** | **1** | **1** | **1** | **1** | **1** | **1** | **1** | **1** | **1** | **1** |
| **144** | **1** | **1** | **1** | **1** | **1** | **1** | **1** | **1** | **1** | **1** |
| **145** | **1** | **1** | **1** | **1** | **1** | **1** | **1** | **1** | **1** | **1** |
| **146** | **1** | **1** | **1** | **1** | **1** | **1** | **1** | **1** | **1** | **1** |
| **147** | **1** | **1** | **1** | **1** | **1** | **1** | **1** | **1** | **1** | **1** |
| **148** | **1** | **1** | **1** | **1** | **1** | **1** | **1** | **1** | **1** | **1** |
| **149** | **1** | **1** | **1** | **1** | **1** | **1** | **1** | **1** | **1** | **1** |
| **150** | **1** | **1** | **1** | **1** | **1** | **1** | **1** | **1** | **1** | **1** |
| **151** | **0** | **0** | **0** | **0** | **0** | **0** | **0** | **0** | **0** | **0** |
| **152** | **0** | **0** | **0** | **0** | **0** | **0** | **0** | **0** | **0** | **0** |
| **153** | **0** | **0** | **0** | **0** | **0** | **0** | **0** | **0** | **0** | **0** |
| **154** | **1** | **1** | **0** | **0** | **1** | **0** | **0** | **0** | **1** | **0** |
| **155** | **1** | **1** | **1** | **1** | **1** | **1** | **1** | **1** | **1** | **1** |
| **156** | **1** | **1** | **1** | **1** | **1** | **1** | **1** | **1** | **1** | **1** |
| **157** | **1** | **1** | **1** | **1** | **1** | **1** | **1** | **1** | **1** | **1** |
| **158** | **1** | **1** | **1** | **1** | **1** | **1** | **1** | **1** | **1** | **1** |
| **159** | **0** | **0** | **0** | **0** | **0** | **0** | **0** | **0** | **0** | **0** |
| **160** | **1** | **1** | **1** | **1** | **1** | **1** | **1** | **1** | **1** | **1** |
| **161** | **1** | **1** | **1** | **1** | **1** | **1** | **1** | **1** | **1** | **1** |
| **162** | **1** | **1** | **1** | **1** | **1** | **1** | **1** | **1** | **1** | **1** |
| **163** | **1** | **1** | **1** | **1** | **1** | **1** | **1** | **1** | **1** | **1** |
| **164** | **1** | **1** | **1** | **1** | **1** | **1** | **1** | **1** | **1** | **1** |
| **165** | **1** | **1** | **1** | **1** | **1** | **1** | **1** | **1** | **1** | **1** |
| **166** | **1** | **1** | **1** | **1** | **1** | **1** | **1** | **1** | **1** | **1** |
| **167** | **1** | **1** | **1** | **1** | **1** | **1** | **1** | **1** | **1** | **1** |
| **168** | **1** | **1** | **1** | **1** | **1** | **1** | **1** | **1** | **1** | **1** |
| **169** | **1** | **1** | **1** | **1** | **1** | **1** | **1** | **1** | **1** | **1** |
| **170** | **1** | **1** | **1** | **1** | **1** | **1** | **1** | **1** | **1** | **1** |
| **171** | **0** | **0** | **0** | **0** | **0** | **0** | **0** | **0** | **0** | **0** |
| **172** | **0** | **0** | **0** | **0** | **0** | **0** | **0** | **0** | **0** | **0** |
| **173** | **0** | **0** | **0** | **0** | **0** | **0** | **0** | **0** | **0** | **0** |
| **174** | **1** | **1** | **1** | **1** | **1** | **1** | **1** | **1** | **1** | **1** |
| **175** | **1** | **1** | **1** | **1** | **1** | **1** | **1** | **1** | **1** | **1** |
| **176** | **1** | **1** | **1** | **1** | **1** | **1** | **1** | **1** | **1** | **1** |
| **177** | **1** | **1** | **1** | **1** | **1** | **1** | **1** | **1** | **1** | **1** |
| **178** | **1** | **1** | **1** | **1** | **1** | **1** | **1** | **1** | **0** | **1** |
| **179** | **0** | **0** | **0** | **0** | **0** | **0** | **0** | **0** | **0** | **0** |
| **180** | **0** | **0** | **0** | **0** | **0** | **0** | **0** | **0** | **0** | **0** |
| **181** | **1** | **1** | **1** | **1** | **1** | **1** | **1** | **1** | **1** | **1** |
| **182** | **1** | **1** | **1** | **1** | **1** | **1** | **1** | **1** | **1** | **1** |
| **183** | **1** | **1** | **1** | **1** | **1** | **1** | **1** | **1** | **1** | **1** |
| **184** | **1** | **1** | **1** | **1** | **1** | **1** | **1** | **1** | **1** | **1** |
| **185** | **0** | **0** | **0** | **0** | **0** | **0** | **0** | **0** | **0** | **0** |
| **186** | **0** | **0** | **0** | **0** | **0** | **0** | **0** | **0** | **0** | **0** |
| **187** | **0** | **0** | **0** | **0** | **0** | **0** | **0** | **0** | **0** | **0** |
| **188** | **1** | **1** | **1** | **1** | **1** | **1** | **1** | **1** | **1** | **1** |
| **189** | **1** | **1** | **1** | **1** | **1** | **1** | **1** | **1** | **1** | **1** |
| **190** | **1** | **1** | **1** | **1** | **1** | **1** | **1** | **1** | **1** | **1** |
| **191** | **1** | **1** | **1** | **1** | **1** | **1** | **1** | **1** | **1** | **1** |
| **192** | **0** | **0** | **0** | **0** | **0** | **0** | **0** | **0** | **0** | **0** |
| **193** | **1** | **1** | **1** | **1** | **1** | **1** | **1** | **1** | **1** | **1** |
| **194** | **0** | **0** | **0** | **0** | **0** | **0** | **0** | **0** | **0** | **0** |
| **195** | **1** | **1** | **1** | **1** | **1** | **1** | **1** | **1** | **1** | **1** |
| **196** | **0** | **0** | **0** | **1** | **0** | **1** | **0** | **0** | **1** | **0** |
| **197** | **1** | **1** | **1** | **1** | **1** | **1** | **1** | **1** | **1** | **1** |
| **198** | **0** | **0** | **0** | **0** | **0** | **0** | **0** | **0** | **0** | **0** |
| **199** | **1** | **1** | **1** | **1** | **1** | **1** | **1** | **1** | **1** | **1** |
| **200** | **0** | **0** | **0** | **0** | **0** | **0** | **0** | **0** | **0** | **0** |

| **ID** | **UroBot_3.5_1** | **UroBot_3.5_2** | **UroBot_3.5_3** | **UroBot_3.5_4** | **UroBot_3.5_5** | **UroBot_3.5_6** | **UroBot_3.5_7** | **UroBot_3.5_8** | **UroBot_3.5_9** | **UroBot_3.5_10** |
| --- | --- | --- | --- | --- | --- | --- | --- | --- | --- | --- |
| **1** | **1** | **1** | **1** | **1** | **1** | **1** | **1** | **1** | **1** | **1** |
| **2** | **1** | **1** | **1** | **1** | **1** | **1** | **1** | **1** | **1** | **1** |
| **3** | **1** | **1** | **1** | **1** | **1** | **1** | **1** | **1** | **1** | **1** |
| **4** | **1** | **1** | **1** | **1** | **1** | **1** | **1** | **1** | **1** | **1** |
| **5** | **1** | **1** | **1** | **1** | **1** | **1** | **1** | **1** | **1** | **1** |
| **6** | **1** | **1** | **1** | **1** | **1** | **1** | **1** | **1** | **1** | **1** |
| **7** | **0** | **0** | **0** | **0** | **0** | **0** | **0** | **0** | **0** | **0** |
| **8** | **1** | **1** | **1** | **1** | **1** | **1** | **1** | **1** | **1** | **1** |
| **9** | **1** | **1** | **1** | **1** | **1** | **1** | **1** | **1** | **1** | **1** |
| **10** | **1** | **1** | **1** | **1** | **1** | **1** | **1** | **1** | **1** | **1** |
| **11** | **0** | **0** | **0** | **0** | **0** | **0** | **0** | **0** | **0** | **0** |
| **12** | **0** | **0** | **0** | **0** | **0** | **0** | **0** | **0** | **0** | **0** |
| **13** | **0** | **0** | **0** | **0** | **0** | **0** | **0** | **0** | **0** | **0** |
| **14** | **1** | **1** | **1** | **1** | **1** | **1** | **1** | **1** | **1** | **1** |
| **15** | **1** | **1** | **1** | **1** | **1** | **1** | **1** | **1** | **1** | **1** |
| **16** | **1** | **1** | **1** | **1** | **1** | **1** | **1** | **1** | **1** | **1** |
| **17** | **1** | **1** | **1** | **1** | **1** | **1** | **1** | **1** | **1** | **1** |
| **18** | **1** | **1** | **1** | **1** | **1** | **1** | **1** | **1** | **1** | **1** |
| **19** | **0** | **0** | **0** | **0** | **0** | **0** | **0** | **0** | **0** | **0** |
| **20** | **1** | **1** | **1** | **1** | **1** | **1** | **1** | **1** | **1** | **1** |
| **21** | **1** | **1** | **1** | **1** | **1** | **1** | **1** | **1** | **1** | **1** |
| **22** | **0** | **0** | **0** | **0** | **0** | **0** | **0** | **0** | **0** | **0** |
| **23** | **1** | **1** | **1** | **1** | **1** | **1** | **1** | **1** | **1** | **1** |
| **24** | **0** | **0** | **0** | **0** | **0** | **0** | **0** | **0** | **0** | **0** |
| **25** | **1** | **1** | **1** | **1** | **1** | **1** | **1** | **1** | **1** | **1** |
| **26** | **0** | **0** | **0** | **0** | **0** | **0** | **1** | **0** | **1** | **0** |
| **27** | **0** | **0** | **0** | **0** | **0** | **0** | **0** | **0** | **0** | **0** |
| **28** | **1** | **1** | **1** | **1** | **1** | **1** | **1** | **1** | **1** | **1** |
| **29** | **0** | **0** | **0** | **0** | **0** | **0** | **0** | **0** | **0** | **0** |
| **30** | **1** | **1** | **1** | **1** | **1** | **1** | **1** | **1** | **1** | **1** |
| **31** | **1** | **1** | **1** | **1** | **1** | **1** | **1** | **1** | **1** | **1** |
| **32** | **1** | **1** | **1** | **1** | **1** | **1** | **1** | **1** | **1** | **1** |
| **33** | **1** | **1** | **1** | **1** | **1** | **1** | **1** | **1** | **1** | **1** |
| **34** | **1** | **1** | **1** | **1** | **1** | **1** | **1** | **1** | **1** | **1** |
| **35** | **1** | **1** | **1** | **1** | **1** | **1** | **1** | **1** | **1** | **1** |
| **36** | **1** | **1** | **1** | **1** | **1** | **1** | **1** | **1** | **1** | **1** |
| **37** | **0** | **0** | **0** | **0** | **0** | **0** | **0** | **0** | **0** | **0** |
| **38** | **0** | **0** | **0** | **0** | **0** | **0** | **0** | **0** | **0** | **0** |
| **39** | **1** | **1** | **1** | **1** | **1** | **1** | **1** | **1** | **1** | **1** |
| **40** | **1** | **1** | **1** | **1** | **1** | **1** | **1** | **1** | **1** | **1** |
| **41** | **0** | **0** | **0** | **0** | **0** | **0** | **0** | **0** | **0** | **0** |
| **42** | **1** | **1** | **1** | **1** | **1** | **1** | **1** | **1** | **1** | **1** |
| **43** | **0** | **0** | **0** | **0** | **0** | **0** | **0** | **0** | **0** | **0** |
| **44** | **1** | **1** | **1** | **1** | **1** | **1** | **1** | **1** | **1** | **1** |
| **45** | **1** | **1** | **1** | **1** | **1** | **1** | **1** | **1** | **1** | **1** |
| **46** | **1** | **1** | **1** | **1** | **1** | **1** | **1** | **1** | **1** | **1** |
| **47** | **0** | **1** | **0** | **1** | **1** | **1** | **1** | **1** | **0** | **0** |
| **48** | **0** | **0** | **0** | **0** | **0** | **0** | **0** | **0** | **0** | **0** |
| **49** | **1** | **1** | **1** | **1** | **1** | **1** | **1** | **1** | **1** | **1** |
| **50** | **1** | **1** | **1** | **1** | **1** | **1** | **1** | **1** | **1** | **1** |
| **51** | **0** | **0** | **0** | **0** | **0** | **0** | **0** | **0** | **0** | **0** |
| **52** | **1** | **1** | **1** | **1** | **1** | **1** | **1** | **1** | **1** | **1** |
| **53** | **1** | **1** | **1** | **1** | **1** | **1** | **0** | **1** | **1** | **1** |
| **54** | **0** | **0** | **0** | **0** | **0** | **0** | **0** | **0** | **0** | **0** |
| **55** | **1** | **1** | **1** | **1** | **1** | **1** | **1** | **1** | **1** | **1** |
| **56** | **1** | **1** | **1** | **1** | **1** | **1** | **1** | **1** | **1** | **1** |
| **57** | **0** | **0** | **0** | **0** | **0** | **0** | **0** | **0** | **0** | **0** |
| **58** | **1** | **1** | **1** | **1** | **1** | **1** | **1** | **1** | **1** | **1** |
| **59** | **1** | **1** | **1** | **1** | **1** | **1** | **1** | **1** | **1** | **1** |
| **60** | **1** | **1** | **0** | **1** | **0** | **0** | **0** | **0** | **1** | **0** |
| **61** | **0** | **0** | **0** | **0** | **0** | **0** | **0** | **0** | **0** | **0** |
| **62** | **1** | **1** | **1** | **1** | **1** | **1** | **1** | **1** | **1** | **1** |
| **63** | **1** | **1** | **1** | **1** | **1** | **1** | **1** | **1** | **1** | **1** |
| **64** | **1** | **1** | **1** | **1** | **1** | **1** | **1** | **1** | **1** | **1** |
| **65** | **1** | **1** | **1** | **1** | **1** | **1** | **1** | **1** | **1** | **1** |
| **66** | **0** | **0** | **0** | **0** | **0** | **0** | **0** | **0** | **0** | **0** |
| **67** | **1** | **1** | **1** | **1** | **1** | **1** | **1** | **1** | **1** | **1** |
| **68** | **0** | **0** | **1** | **1** | **1** | **1** | **1** | **1** | **1** | **1** |
| **69** | **1** | **1** | **1** | **1** | **1** | **1** | **1** | **1** | **1** | **1** |
| **70** | **1** | **1** | **1** | **1** | **1** | **1** | **1** | **1** | **1** | **1** |
| **71** | **0** | **0** | **0** | **0** | **0** | **0** | **0** | **0** | **0** | **0** |
| **72** | **0** | **0** | **1** | **0** | **0** | **0** | **0** | **0** | **0** | **0** |
| **73** | **1** | **1** | **1** | **1** | **1** | **1** | **1** | **1** | **1** | **1** |
| **74** | **1** | **1** | **1** | **1** | **1** | **1** | **1** | **1** | **1** | **1** |
| **75** | **1** | **1** | **1** | **1** | **1** | **1** | **1** | **1** | **1** | **1** |
| **76** | **1** | **1** | **1** | **1** | **1** | **1** | **1** | **1** | **1** | **1** |
| **77** | **0** | **0** | **1** | **0** | **0** | **0** | **0** | **1** | **0** | **0** |
| **78** | **1** | **1** | **1** | **1** | **1** | **1** | **1** | **1** | **1** | **1** |
| **79** | **1** | **1** | **1** | **1** | **1** | **1** | **1** | **1** | **1** | **1** |
| **80** | **0** | **0** | **0** | **0** | **0** | **0** | **0** | **0** | **0** | **0** |
| **81** | **1** | **1** | **1** | **1** | **0** | **1** | **0** | **1** | **1** | **1** |
| **82** | **0** | **0** | **0** | **0** | **0** | **0** | **0** | **0** | **0** | **0** |
| **83** | **1** | **1** | **1** | **1** | **1** | **1** | **1** | **1** | **1** | **1** |
| **84** | **0** | **0** | **0** | **0** | **0** | **0** | **0** | **0** | **0** | **0** |
| **85** | **0** | **0** | **0** | **0** | **0** | **0** | **0** | **0** | **0** | **0** |
| **86** | **0** | **1** | **1** | **1** | **1** | **1** | **1** | **0** | **1** | **0** |
| **87** | **0** | **0** | **0** | **0** | **0** | **0** | **0** | **0** | **0** | **0** |
| **88** | **1** | **1** | **1** | **1** | **1** | **1** | **1** | **1** | **1** | **1** |
| **89** | **1** | **1** | **1** | **1** | **1** | **1** | **1** | **1** | **1** | **1** |
| **90** | **1** | **1** | **1** | **1** | **1** | **1** | **1** | **1** | **1** | **1** |
| **91** | **1** | **1** | **1** | **1** | **1** | **1** | **1** | **1** | **1** | **1** |
| **92** | **1** | **1** | **1** | **1** | **1** | **1** | **1** | **1** | **1** | **1** |
| **93** | **1** | **1** | **1** | **1** | **1** | **1** | **1** | **1** | **1** | **1** |
| **94** | **1** | **1** | **1** | **1** | **1** | **1** | **1** | **1** | **1** | **1** |
| **95** | **1** | **1** | **1** | **1** | **1** | **1** | **1** | **1** | **1** | **1** |
| **96** | **1** | **1** | **1** | **1** | **1** | **1** | **1** | **1** | **1** | **1** |
| **97** | **1** | **1** | **1** | **1** | **1** | **1** | **1** | **1** | **1** | **1** |
| **98** | **1** | **1** | **1** | **1** | **1** | **1** | **1** | **1** | **1** | **1** |
| **99** | **1** | **1** | **1** | **1** | **1** | **1** | **1** | **1** | **1** | **0** |
| **100** | **1** | **1** | **1** | **1** | **1** | **1** | **1** | **1** | **1** | **1** |
| **101** | **1** | **1** | **1** | **1** | **1** | **1** | **1** | **1** | **1** | **1** |
| **102** | **1** | **1** | **1** | **1** | **1** | **1** | **1** | **1** | **1** | **1** |
| **103** | **0** | **0** | **0** | **0** | **0** | **0** | **0** | **0** | **0** | **0** |
| **104** | **1** | **1** | **1** | **1** | **1** | **1** | **1** | **1** | **1** | **1** |
| **105** | **1** | **1** | **1** | **1** | **1** | **1** | **1** | **1** | **1** | **1** |
| **106** | **1** | **1** | **1** | **1** | **1** | **1** | **1** | **1** | **1** | **1** |
| **107** | **1** | **1** | **1** | **1** | **1** | **1** | **1** | **1** | **1** | **1** |
| **108** | **0** | **0** | **0** | **0** | **0** | **0** | **0** | **0** | **0** | **0** |
| **109** | **1** | **1** | **1** | **1** | **1** | **1** | **1** | **1** | **1** | **1** |
| **110** | **0** | **0** | **0** | **0** | **1** | **0** | **0** | **1** | **0** | **0** |
| **111** | **1** | **1** | **1** | **1** | **1** | **1** | **1** | **1** | **1** | **1** |
| **112** | **1** | **1** | **1** | **1** | **1** | **1** | **1** | **1** | **1** | **1** |
| **113** | **1** | **1** | **1** | **1** | **1** | **1** | **1** | **1** | **1** | **1** |
| **114** | **1** | **1** | **1** | **1** | **1** | **1** | **1** | **1** | **1** | **1** |
| **115** | **0** | **0** | **0** | **0** | **0** | **0** | **0** | **0** | **0** | **0** |
| **116** | **1** | **1** | **1** | **1** | **1** | **1** | **1** | **1** | **1** | **1** |
| **117** | **1** | **1** | **1** | **1** | **1** | **1** | **1** | **1** | **1** | **1** |
| **118** | **1** | **1** | **1** | **1** | **1** | **1** | **1** | **1** | **1** | **1** |
| **119** | **1** | **1** | **0** | **1** | **1** | **1** | **1** | **1** | **1** | **1** |
| **120** | **0** | **1** | **0** | **1** | **0** | **0** | **0** | **0** | **0** | **1** |
| **121** | **1** | **1** | **1** | **1** | **1** | **1** | **1** | **1** | **1** | **1** |
| **122** | **0** | **0** | **0** | **0** | **0** | **0** | **0** | **0** | **0** | **0** |
| **123** | **1** | **1** | **1** | **1** | **1** | **1** | **1** | **1** | **1** | **1** |
| **124** | **1** | **1** | **1** | **1** | **1** | **1** | **1** | **1** | **1** | **1** |
| **125** | **1** | **1** | **1** | **1** | **1** | **1** | **1** | **1** | **1** | **1** |
| **126** | **1** | **1** | **1** | **1** | **1** | **1** | **1** | **1** | **1** | **1** |
| **127** | **0** | **0** | **0** | **0** | **0** | **0** | **0** | **0** | **0** | **0** |
| **128** | **1** | **1** | **1** | **1** | **1** | **1** | **1** | **1** | **1** | **1** |
| **129** | **1** | **1** | **1** | **1** | **1** | **1** | **1** | **1** | **1** | **1** |
| **130** | **1** | **1** | **1** | **1** | **1** | **1** | **1** | **1** | **1** | **1** |
| **131** | **1** | **1** | **1** | **1** | **1** | **1** | **1** | **1** | **1** | **1** |
| **132** | **0** | **0** | **0** | **0** | **0** | **0** | **0** | **0** | **0** | **0** |
| **133** | **1** | **1** | **1** | **1** | **1** | **1** | **1** | **1** | **1** | **1** |
| **134** | **1** | **1** | **1** | **1** | **1** | **1** | **1** | **1** | **1** | **1** |
| **135** | **1** | **1** | **1** | **1** | **1** | **1** | **1** | **1** | **1** | **1** |
| **136** | **1** | **1** | **1** | **1** | **1** | **1** | **1** | **1** | **1** | **1** |
| **137** | **0** | **0** | **0** | **0** | **0** | **0** | **0** | **0** | **0** | **0** |
| **138** | **1** | **1** | **1** | **1** | **1** | **1** | **1** | **1** | **1** | **1** |
| **139** | **0** | **0** | **0** | **0** | **0** | **0** | **0** | **0** | **0** | **0** |
| **140** | **1** | **1** | **1** | **1** | **1** | **1** | **1** | **1** | **0** | **1** |
| **141** | **1** | **0** | **0** | **0** | **0** | **0** | **0** | **0** | **0** | **0** |
| **142** | **1** | **1** | **1** | **1** | **1** | **1** | **1** | **1** | **1** | **1** |
| **143** | **1** | **1** | **1** | **1** | **1** | **1** | **1** | **1** | **1** | **1** |
| **144** | **1** | **1** | **1** | **1** | **1** | **1** | **1** | **1** | **1** | **1** |
| **145** | **1** | **1** | **1** | **1** | **1** | **1** | **1** | **1** | **1** | **1** |
| **146** | **0** | **0** | **0** | **0** | **0** | **0** | **0** | **0** | **0** | **0** |
| **147** | **1** | **1** | **1** | **1** | **1** | **1** | **1** | **1** | **1** | **1** |
| **148** | **1** | **1** | **1** | **1** | **1** | **1** | **1** | **1** | **1** | **1** |
| **149** | **1** | **1** | **1** | **1** | **1** | **1** | **1** | **1** | **1** | **1** |
| **150** | **0** | **0** | **0** | **0** | **0** | **0** | **0** | **0** | **0** | **0** |
| **151** | **1** | **1** | **1** | **1** | **1** | **1** | **1** | **1** | **1** | **1** |
| **152** | **1** | **0** | **0** | **0** | **0** | **0** | **0** | **0** | **0** | **0** |
| **153** | **0** | **0** | **0** | **0** | **0** | **0** | **0** | **0** | **0** | **0** |
| **154** | **0** | **1** | **1** | **0** | **1** | **1** | **0** | **1** | **0** | **0** |
| **155** | **0** | **0** | **1** | **0** | **0** | **0** | **0** | **0** | **0** | **0** |
| **156** | **1** | **1** | **1** | **1** | **1** | **1** | **1** | **1** | **1** | **1** |
| **157** | **1** | **1** | **1** | **1** | **1** | **1** | **1** | **1** | **1** | **1** |
| **158** | **1** | **1** | **0** | **1** | **0** | **1** | **1** | **1** | **1** | **1** |
| **159** | **1** | **1** | **1** | **1** | **1** | **1** | **1** | **1** | **1** | **1** |
| **160** | **1** | **1** | **1** | **1** | **1** | **1** | **1** | **1** | **1** | **1** |
| **161** | **1** | **1** | **1** | **1** | **1** | **1** | **1** | **1** | **1** | **1** |
| **162** | **1** | **1** | **1** | **1** | **1** | **1** | **1** | **1** | **1** | **1** |
| **163** | **1** | **1** | **1** | **1** | **1** | **1** | **1** | **1** | **1** | **1** |
| **164** | **1** | **1** | **1** | **1** | **1** | **1** | **1** | **1** | **1** | **1** |
| **165** | **0** | **0** | **0** | **0** | **0** | **0** | **0** | **0** | **0** | **0** |
| **166** | **1** | **1** | **1** | **1** | **1** | **1** | **1** | **1** | **1** | **1** |
| **167** | **1** | **1** | **1** | **1** | **1** | **1** | **1** | **1** | **1** | **1** |
| **168** | **1** | **1** | **1** | **1** | **1** | **1** | **1** | **1** | **1** | **1** |
| **169** | **1** | **1** | **1** | **1** | **1** | **1** | **1** | **1** | **1** | **1** |
| **170** | **1** | **1** | **1** | **1** | **1** | **1** | **1** | **1** | **1** | **1** |
| **171** | **1** | **1** | **1** | **1** | **1** | **1** | **1** | **1** | **1** | **1** |
| **172** | **0** | **0** | **0** | **0** | **0** | **0** | **0** | **0** | **0** | **0** |
| **173** | **1** | **1** | **1** | **1** | **1** | **1** | **1** | **1** | **1** | **1** |
| **174** | **1** | **1** | **0** | **1** | **0** | **0** | **0** | **0** | **0** | **0** |
| **175** | **1** | **1** | **1** | **1** | **1** | **1** | **1** | **1** | **1** | **1** |
| **176** | **1** | **1** | **1** | **1** | **1** | **1** | **1** | **1** | **1** | **1** |
| **177** | **1** | **0** | **0** | **0** | **1** | **0** | **1** | **0** | **1** | **0** |
| **178** | **0** | **0** | **0** | **0** | **0** | **0** | **0** | **0** | **0** | **0** |
| **179** | **1** | **1** | **0** | **0** | **0** | **0** | **0** | **1** | **0** | **1** |
| **180** | **1** | **1** | **1** | **1** | **1** | **1** | **1** | **1** | **1** | **1** |
| **181** | **0** | **0** | **0** | **0** | **0** | **0** | **0** | **0** | **0** | **0** |
| **182** | **1** | **1** | **1** | **1** | **1** | **1** | **1** | **1** | **1** | **1** |
| **183** | **1** | **1** | **1** | **1** | **1** | **1** | **1** | **1** | **1** | **1** |
| **184** | **1** | **1** | **1** | **1** | **1** | **1** | **1** | **1** | **1** | **1** |
| **185** | **1** | **1** | **1** | **1** | **1** | **1** | **1** | **1** | **1** | **1** |
| **186** | **1** | **1** | **1** | **1** | **1** | **1** | **1** | **1** | **1** | **1** |
| **187** | **1** | **1** | **1** | **1** | **1** | **1** | **1** | **1** | **1** | **1** |
| **188** | **1** | **1** | **1** | **0** | **1** | **1** | **1** | **1** | **1** | **1** |
| **189** | **0** | **0** | **0** | **0** | **0** | **0** | **0** | **0** | **0** | **0** |
| **190** | **1** | **1** | **1** | **1** | **1** | **1** | **1** | **1** | **1** | **1** |
| **191** | **1** | **1** | **1** | **1** | **1** | **1** | **1** | **1** | **1** | **1** |
| **192** | **0** | **0** | **0** | **0** | **0** | **0** | **0** | **0** | **0** | **0** |
| **193** | **1** | **1** | **1** | **1** | **1** | **1** | **1** | **1** | **1** | **1** |
| **194** | **1** | **1** | **1** | **1** | **1** | **1** | **1** | **1** | **1** | **1** |
| **195** | **1** | **1** | **1** | **1** | **1** | **1** | **1** | **1** | **1** | **1** |
| **196** | **1** | **1** | **1** | **1** | **1** | **1** | **1** | **1** | **1** | **1** |
| **197** | **1** | **1** | **1** | **1** | **1** | **1** | **1** | **1** | **1** | **1** |
| **198** | **0** | **0** | **0** | **0** | **0** | **0** | **0** | **0** | **0** | **0** |
| **199** | **1** | **1** | **1** | **1** | **1** | **1** | **1** | **1** | **1** | **1** |
| **200** | **0** | **0** | **0** | **0** | **0** | **0** | **0** | **0** | **0** | **0** |

| **ID** | **UroBot_4_1** | **UroBot_4_2** | **UroBot_4_3** | **UroBot_4_4** | **UroBot_4_5** | **UroBot_4_6** | **UroBot_4_7** | **UroBot_4_8** | **UroBot_4_9** | **UroBot_4_10** |
| --- | --- | --- | --- | --- | --- | --- | --- | --- | --- | --- |
| **1** | **1** | **1** | **1** | **1** | **1** | **1** | **1** | **1** | **1** | **1** |
| **2** | **1** | **1** | **1** | **1** | **1** | **1** | **1** | **1** | **1** | **1** |
| **3** | **1** | **1** | **1** | **1** | **1** | **1** | **1** | **1** | **1** | **1** |
| **4** | **1** | **1** | **1** | **1** | **1** | **1** | **1** | **1** | **1** | **1** |
| **5** | **1** | **1** | **1** | **1** | **1** | **1** | **1** | **1** | **1** | **1** |
| **6** | **1** | **1** | **1** | **1** | **1** | **1** | **1** | **1** | **1** | **1** |
| **7** | **1** | **1** | **1** | **1** | **1** | **1** | **1** | **1** | **1** | **1** |
| **8** | **1** | **1** | **1** | **1** | **1** | **1** | **1** | **1** | **1** | **1** |
| **9** | **1** | **1** | **1** | **1** | **1** | **1** | **1** | **1** | **1** | **1** |
| **10** | **1** | **1** | **1** | **1** | **1** | **1** | **1** | **1** | **1** | **1** |
| **11** | **1** | **1** | **1** | **1** | **1** | **1** | **1** | **1** | **1** | **1** |
| **12** | **0** | **0** | **0** | **0** | **0** | **0** | **0** | **0** | **0** | **0** |
| **13** | **0** | **0** | **0** | **0** | **0** | **0** | **0** | **0** | **0** | **0** |
| **14** | **1** | **1** | **1** | **1** | **1** | **1** | **1** | **1** | **1** | **1** |
| **15** | **1** | **1** | **1** | **1** | **1** | **1** | **1** | **1** | **1** | **1** |
| **16** | **1** | **1** | **1** | **1** | **1** | **1** | **1** | **1** | **1** | **1** |
| **17** | **1** | **1** | **1** | **1** | **1** | **1** | **1** | **1** | **1** | **1** |
| **18** | **1** | **1** | **1** | **1** | **1** | **1** | **1** | **1** | **1** | **1** |
| **19** | **1** | **1** | **1** | **1** | **1** | **1** | **1** | **1** | **1** | **1** |
| **20** | **1** | **1** | **1** | **1** | **1** | **1** | **1** | **1** | **1** | **1** |
| **21** | **1** | **1** | **1** | **1** | **1** | **1** | **1** | **1** | **1** | **1** |
| **22** | **0** | **0** | **0** | **0** | **0** | **0** | **0** | **0** | **0** | **0** |
| **23** | **1** | **1** | **1** | **1** | **1** | **1** | **1** | **1** | **1** | **1** |
| **24** | **0** | **0** | **0** | **0** | **0** | **0** | **0** | **0** | **0** | **0** |
| **25** | **1** | **1** | **1** | **1** | **1** | **1** | **1** | **1** | **1** | **1** |
| **26** | **1** | **1** | **1** | **1** | **1** | **1** | **1** | **1** | **1** | **1** |
| **27** | **1** | **1** | **1** | **1** | **1** | **1** | **1** | **1** | **1** | **1** |
| **28** | **1** | **1** | **1** | **1** | **1** | **1** | **1** | **1** | **1** | **1** |
| **29** | **0** | **0** | **0** | **0** | **1** | **0** | **0** | **0** | **1** | **0** |
| **30** | **1** | **1** | **1** | **1** | **1** | **1** | **1** | **1** | **1** | **1** |
| **31** | **1** | **1** | **1** | **1** | **1** | **1** | **1** | **1** | **1** | **1** |
| **32** | **1** | **1** | **1** | **1** | **1** | **1** | **1** | **1** | **1** | **1** |
| **33** | **1** | **1** | **1** | **1** | **1** | **1** | **1** | **1** | **1** | **1** |
| **34** | **1** | **1** | **1** | **1** | **1** | **1** | **1** | **1** | **1** | **1** |
| **35** | **1** | **1** | **1** | **1** | **1** | **1** | **1** | **1** | **1** | **1** |
| **36** | **1** | **1** | **1** | **1** | **1** | **1** | **1** | **1** | **1** | **1** |
| **37** | **0** | **0** | **0** | **0** | **0** | **0** | **0** | **0** | **0** | **0** |
| **38** | **0** | **0** | **0** | **0** | **0** | **0** | **0** | **0** | **0** | **0** |
| **39** | **1** | **1** | **1** | **1** | **1** | **1** | **1** | **1** | **1** | **1** |
| **40** | **1** | **1** | **1** | **1** | **1** | **1** | **1** | **1** | **1** | **1** |
| **41** | **0** | **0** | **0** | **0** | **0** | **0** | **0** | **0** | **0** | **0** |
| **42** | **1** | **1** | **1** | **1** | **1** | **1** | **1** | **1** | **1** | **1** |
| **43** | **1** | **1** | **1** | **1** | **1** | **1** | **1** | **1** | **1** | **1** |
| **44** | **1** | **1** | **1** | **1** | **1** | **1** | **1** | **1** | **1** | **1** |
| **45** | **1** | **1** | **1** | **1** | **1** | **1** | **1** | **1** | **1** | **1** |
| **46** | **1** | **1** | **1** | **1** | **1** | **1** | **1** | **1** | **1** | **1** |
| **47** | **1** | **1** | **1** | **1** | **1** | **1** | **1** | **1** | **1** | **1** |
| **48** | **1** | **1** | **1** | **1** | **1** | **1** | **1** | **1** | **1** | **1** |
| **49** | **1** | **1** | **1** | **1** | **1** | **1** | **1** | **1** | **1** | **1** |
| **50** | **1** | **1** | **1** | **1** | **1** | **1** | **0** | **1** | **0** | **0** |
| **51** | **0** | **0** | **1** | **1** | **1** | **1** | **1** | **1** | **0** | **1** |
| **52** | **1** | **1** | **1** | **1** | **1** | **1** | **1** | **1** | **1** | **1** |
| **53** | **0** | **1** | **0** | **1** | **0** | **1** | **0** | **0** | **0** | **0** |
| **54** | **1** | **1** | **1** | **1** | **1** | **1** | **1** | **1** | **1** | **1** |
| **55** | **1** | **1** | **1** | **1** | **1** | **1** | **1** | **1** | **1** | **1** |
| **56** | **1** | **1** | **1** | **1** | **1** | **1** | **1** | **1** | **1** | **1** |
| **57** | **1** | **1** | **1** | **1** | **1** | **1** | **1** | **1** | **1** | **1** |
| **58** | **1** | **1** | **1** | **1** | **1** | **1** | **1** | **1** | **1** | **1** |
| **59** | **1** | **1** | **1** | **1** | **1** | **1** | **1** | **1** | **1** | **1** |
| **60** | **1** | **1** | **1** | **1** | **1** | **1** | **1** | **1** | **1** | **1** |
| **61** | **0** | **0** | **0** | **0** | **0** | **0** | **0** | **0** | **0** | **0** |
| **62** | **1** | **1** | **1** | **1** | **1** | **1** | **1** | **1** | **1** | **1** |
| **63** | **1** | **1** | **1** | **1** | **1** | **1** | **1** | **1** | **1** | **1** |
| **64** | **1** | **1** | **1** | **1** | **1** | **1** | **1** | **1** | **1** | **1** |
| **65** | **1** | **1** | **1** | **1** | **1** | **1** | **1** | **1** | **1** | **1** |
| **66** | **1** | **1** | **1** | **1** | **1** | **1** | **1** | **1** | **1** | **1** |
| **67** | **1** | **1** | **1** | **1** | **1** | **1** | **1** | **1** | **1** | **1** |
| **68** | **0** | **0** | **0** | **0** | **0** | **0** | **0** | **0** | **0** | **0** |
| **69** | **1** | **1** | **1** | **1** | **1** | **1** | **1** | **1** | **1** | **1** |
| **70** | **1** | **1** | **1** | **1** | **1** | **1** | **1** | **1** | **1** | **1** |
| **71** | **1** | **1** | **1** | **1** | **1** | **1** | **1** | **1** | **1** | **1** |
| **72** | **1** | **1** | **1** | **1** | **1** | **1** | **1** | **1** | **1** | **1** |
| **73** | **1** | **1** | **1** | **1** | **1** | **1** | **1** | **1** | **1** | **1** |
| **74** | **1** | **1** | **1** | **1** | **1** | **1** | **1** | **1** | **1** | **1** |
| **75** | **1** | **1** | **1** | **1** | **1** | **1** | **1** | **1** | **1** | **1** |
| **76** | **1** | **1** | **1** | **1** | **1** | **1** | **1** | **1** | **1** | **1** |
| **77** | **1** | **1** | **1** | **0** | **1** | **1** | **1** | **0** | **1** | **0** |
| **78** | **1** | **1** | **1** | **1** | **1** | **1** | **1** | **1** | **1** | **1** |
| **79** | **1** | **1** | **1** | **1** | **1** | **1** | **1** | **1** | **1** | **1** |
| **80** | **0** | **0** | **0** | **0** | **0** | **0** | **0** | **0** | **0** | **0** |
| **81** | **1** | **1** | **1** | **1** | **1** | **1** | **1** | **1** | **1** | **1** |
| **82** | **0** | **0** | **0** | **0** | **0** | **0** | **0** | **0** | **0** | **0** |
| **83** | **1** | **1** | **1** | **1** | **1** | **1** | **1** | **1** | **1** | **1** |
| **84** | **0** | **0** | **0** | **0** | **0** | **0** | **0** | **0** | **0** | **0** |
| **85** | **0** | **0** | **0** | **0** | **0** | **0** | **0** | **0** | **0** | **0** |
| **86** | **1** | **1** | **1** | **1** | **1** | **1** | **1** | **1** | **1** | **1** |
| **87** | **1** | **1** | **1** | **1** | **1** | **1** | **1** | **1** | **1** | **1** |
| **88** | **1** | **1** | **1** | **1** | **1** | **1** | **1** | **1** | **1** | **1** |
| **89** | **1** | **1** | **1** | **1** | **1** | **1** | **1** | **1** | **1** | **1** |
| **90** | **1** | **1** | **1** | **1** | **1** | **1** | **1** | **1** | **1** | **1** |
| **91** | **1** | **1** | **1** | **1** | **1** | **1** | **1** | **1** | **1** | **1** |
| **92** | **1** | **1** | **1** | **1** | **1** | **1** | **1** | **1** | **1** | **1** |
| **93** | **1** | **1** | **1** | **1** | **1** | **1** | **1** | **1** | **1** | **1** |
| **94** | **1** | **1** | **1** | **1** | **1** | **1** | **1** | **1** | **1** | **1** |
| **95** | **1** | **1** | **1** | **1** | **1** | **1** | **1** | **1** | **1** | **1** |
| **96** | **1** | **1** | **1** | **1** | **1** | **1** | **1** | **1** | **1** | **1** |
| **97** | **1** | **1** | **1** | **1** | **1** | **1** | **1** | **1** | **1** | **1** |
| **98** | **1** | **1** | **1** | **1** | **1** | **1** | **1** | **1** | **1** | **1** |
| **99** | **1** | **1** | **1** | **1** | **1** | **1** | **1** | **1** | **1** | **1** |
| **100** | **1** | **1** | **1** | **1** | **1** | **1** | **1** | **1** | **1** | **1** |
| **101** | **1** | **1** | **1** | **1** | **1** | **1** | **1** | **1** | **1** | **1** |
| **102** | **1** | **1** | **1** | **1** | **1** | **1** | **1** | **1** | **1** | **1** |
| **103** | **1** | **1** | **1** | **1** | **1** | **1** | **1** | **1** | **1** | **1** |
| **104** | **0** | **0** | **0** | **0** | **0** | **0** | **0** | **0** | **0** | **0** |
| **105** | **1** | **1** | **1** | **1** | **1** | **1** | **1** | **1** | **1** | **1** |
| **106** | **1** | **1** | **1** | **1** | **1** | **1** | **1** | **1** | **1** | **1** |
| **107** | **1** | **1** | **1** | **1** | **1** | **1** | **1** | **1** | **1** | **1** |
| **108** | **1** | **1** | **1** | **1** | **1** | **1** | **1** | **1** | **1** | **1** |
| **109** | **1** | **1** | **1** | **1** | **1** | **1** | **1** | **1** | **1** | **1** |
| **110** | **1** | **1** | **1** | **1** | **1** | **1** | **1** | **1** | **1** | **1** |
| **111** | **1** | **1** | **1** | **1** | **1** | **1** | **1** | **1** | **1** | **1** |
| **112** | **1** | **1** | **1** | **1** | **1** | **1** | **1** | **1** | **1** | **1** |
| **113** | **1** | **1** | **1** | **1** | **1** | **1** | **1** | **1** | **1** | **1** |
| **114** | **1** | **1** | **1** | **1** | **1** | **1** | **1** | **1** | **1** | **1** |
| **115** | **0** | **0** | **0** | **0** | **0** | **0** | **0** | **0** | **0** | **0** |
| **116** | **1** | **1** | **1** | **1** | **1** | **1** | **1** | **1** | **1** | **1** |
| **117** | **1** | **1** | **1** | **1** | **0** | **1** | **1** | **1** | **1** | **1** |
| **118** | **1** | **1** | **1** | **1** | **1** | **1** | **1** | **1** | **1** | **1** |
| **119** | **1** | **1** | **1** | **1** | **1** | **1** | **1** | **1** | **1** | **1** |
| **120** | **1** | **1** | **1** | **1** | **1** | **1** | **1** | **1** | **1** | **1** |
| **121** | **1** | **1** | **1** | **1** | **1** | **1** | **1** | **1** | **1** | **1** |
| **122** | **1** | **1** | **1** | **1** | **1** | **1** | **1** | **1** | **1** | **1** |
| **123** | **1** | **1** | **1** | **1** | **1** | **1** | **1** | **1** | **1** | **1** |
| **124** | **1** | **1** | **1** | **1** | **1** | **1** | **1** | **1** | **1** | **1** |
| **125** | **1** | **1** | **1** | **1** | **1** | **1** | **1** | **1** | **1** | **1** |
| **126** | **1** | **1** | **1** | **1** | **1** | **1** | **1** | **1** | **1** | **1** |
| **127** | **1** | **1** | **1** | **1** | **1** | **1** | **1** | **1** | **1** | **1** |
| **128** | **1** | **1** | **1** | **1** | **1** | **1** | **1** | **1** | **1** | **1** |
| **129** | **1** | **1** | **1** | **1** | **1** | **1** | **1** | **1** | **1** | **1** |
| **130** | **1** | **1** | **1** | **1** | **1** | **1** | **1** | **1** | **1** | **1** |
| **131** | **1** | **1** | **1** | **1** | **1** | **1** | **1** | **1** | **1** | **1** |
| **132** | **1** | **1** | **1** | **1** | **1** | **1** | **1** | **1** | **1** | **1** |
| **133** | **1** | **1** | **1** | **1** | **1** | **1** | **1** | **1** | **1** | **1** |
| **134** | **1** | **1** | **1** | **1** | **1** | **1** | **1** | **1** | **1** | **1** |
| **135** | **1** | **1** | **1** | **1** | **1** | **1** | **1** | **1** | **1** | **1** |
| **136** | **1** | **1** | **1** | **1** | **1** | **1** | **1** | **1** | **1** | **1** |
| **137** | **1** | **1** | **1** | **1** | **1** | **1** | **1** | **1** | **1** | **1** |
| **138** | **1** | **1** | **1** | **1** | **1** | **1** | **1** | **1** | **1** | **1** |
| **139** | **0** | **0** | **0** | **0** | **0** | **0** | **0** | **0** | **0** | **0** |
| **140** | **1** | **1** | **1** | **1** | **1** | **1** | **1** | **1** | **1** | **1** |
| **141** | **1** | **1** | **1** | **1** | **1** | **1** | **1** | **1** | **1** | **1** |
| **142** | **1** | **1** | **0** | **0** | **0** | **0** | **0** | **0** | **0** | **1** |
| **143** | **1** | **1** | **1** | **1** | **1** | **1** | **1** | **1** | **1** | **1** |
| **144** | **1** | **1** | **1** | **1** | **1** | **1** | **1** | **1** | **1** | **1** |
| **145** | **1** | **1** | **1** | **1** | **1** | **1** | **1** | **1** | **1** | **1** |
| **146** | **1** | **1** | **1** | **1** | **1** | **1** | **1** | **1** | **1** | **1** |
| **147** | **1** | **1** | **1** | **1** | **1** | **1** | **1** | **1** | **1** | **1** |
| **148** | **1** | **1** | **1** | **1** | **1** | **1** | **1** | **1** | **1** | **1** |
| **149** | **1** | **1** | **1** | **1** | **1** | **1** | **1** | **1** | **1** | **1** |
| **150** | **1** | **1** | **1** | **1** | **1** | **1** | **1** | **1** | **1** | **1** |
| **151** | **1** | **1** | **1** | **1** | **1** | **1** | **1** | **1** | **1** | **1** |
| **152** | **0** | **0** | **0** | **0** | **0** | **0** | **0** | **0** | **0** | **0** |
| **153** | **0** | **0** | **0** | **0** | **0** | **0** | **0** | **0** | **0** | **0** |
| **154** | **1** | **1** | **1** | **1** | **1** | **1** | **1** | **1** | **1** | **1** |
| **155** | **1** | **1** | **1** | **1** | **1** | **1** | **1** | **1** | **1** | **1** |
| **156** | **1** | **1** | **1** | **1** | **1** | **1** | **1** | **1** | **1** | **1** |
| **157** | **1** | **1** | **1** | **1** | **1** | **1** | **1** | **1** | **1** | **1** |
| **158** | **1** | **1** | **1** | **1** | **1** | **1** | **1** | **1** | **1** | **1** |
| **159** | **1** | **1** | **1** | **1** | **1** | **1** | **1** | **1** | **1** | **1** |
| **160** | **1** | **1** | **1** | **1** | **1** | **1** | **1** | **1** | **1** | **1** |
| **161** | **1** | **1** | **1** | **1** | **1** | **1** | **1** | **1** | **1** | **1** |
| **162** | **1** | **1** | **1** | **1** | **1** | **1** | **1** | **1** | **1** | **1** |
| **163** | **1** | **1** | **1** | **1** | **1** | **1** | **1** | **1** | **1** | **1** |
| **164** | **1** | **1** | **1** | **1** | **1** | **1** | **1** | **1** | **1** | **1** |
| **165** | **1** | **1** | **1** | **1** | **1** | **1** | **1** | **1** | **1** | **1** |
| **166** | **1** | **1** | **1** | **1** | **1** | **1** | **1** | **1** | **1** | **1** |
| **167** | **1** | **1** | **1** | **1** | **1** | **1** | **1** | **1** | **1** | **1** |
| **168** | **1** | **1** | **1** | **1** | **1** | **1** | **1** | **1** | **1** | **1** |
| **169** | **1** | **1** | **1** | **1** | **1** | **1** | **1** | **1** | **1** | **1** |
| **170** | **1** | **1** | **1** | **1** | **1** | **1** | **1** | **1** | **1** | **1** |
| **171** | **1** | **1** | **1** | **1** | **1** | **1** | **1** | **1** | **1** | **1** |
| **172** | **0** | **0** | **0** | **0** | **0** | **0** | **0** | **0** | **0** | **0** |
| **173** | **1** | **1** | **1** | **1** | **1** | **1** | **1** | **1** | **1** | **1** |
| **174** | **1** | **1** | **1** | **1** | **1** | **1** | **1** | **1** | **1** | **1** |
| **175** | **1** | **1** | **1** | **1** | **1** | **1** | **1** | **1** | **1** | **1** |
| **176** | **1** | **1** | **1** | **1** | **1** | **1** | **1** | **1** | **1** | **1** |
| **177** | **1** | **1** | **1** | **1** | **1** | **1** | **1** | **1** | **1** | **1** |
| **178** | **1** | **0** | **1** | **1** | **1** | **1** | **1** | **1** | **0** | **1** |
| **179** | **0** | **1** | **0** | **0** | **0** | **0** | **0** | **0** | **0** | **0** |
| **180** | **1** | **1** | **1** | **1** | **1** | **1** | **1** | **1** | **1** | **1** |
| **181** | **1** | **1** | **1** | **1** | **1** | **1** | **1** | **1** | **1** | **1** |
| **182** | **1** | **1** | **1** | **1** | **1** | **1** | **1** | **1** | **1** | **1** |
| **183** | **1** | **1** | **1** | **1** | **1** | **1** | **1** | **1** | **1** | **1** |
| **184** | **1** | **1** | **1** | **1** | **1** | **1** | **1** | **1** | **1** | **1** |
| **185** | **1** | **1** | **1** | **1** | **1** | **1** | **1** | **1** | **1** | **1** |
| **186** | **1** | **1** | **1** | **1** | **1** | **1** | **1** | **1** | **1** | **1** |
| **187** | **1** | **1** | **1** | **1** | **1** | **1** | **1** | **1** | **1** | **1** |
| **188** | **1** | **1** | **1** | **1** | **1** | **1** | **1** | **1** | **1** | **1** |
| **189** | **0** | **0** | **0** | **0** | **0** | **0** | **0** | **0** | **0** | **0** |
| **190** | **1** | **1** | **1** | **1** | **1** | **1** | **1** | **1** | **1** | **1** |
| **191** | **1** | **1** | **1** | **1** | **1** | **1** | **1** | **1** | **1** | **1** |
| **192** | **0** | **0** | **0** | **0** | **0** | **0** | **0** | **0** | **0** | **0** |
| **193** | **0** | **0** | **0** | **0** | **0** | **0** | **0** | **0** | **0** | **0** |
| **194** | **1** | **1** | **1** | **1** | **1** | **1** | **1** | **1** | **1** | **1** |
| **195** | **1** | **1** | **1** | **1** | **1** | **1** | **1** | **1** | **1** | **1** |
| **196** | **1** | **1** | **1** | **1** | **1** | **1** | **1** | **1** | **1** | **1** |
| **197** | **1** | **1** | **1** | **1** | **1** | **1** | **1** | **1** | **1** | **1** |
| **198** | **1** | **1** | **1** | **1** | **1** | **1** | **1** | **1** | **1** | **1** |
| **199** | **1** | **1** | **1** | **1** | **1** | **1** | **1** | **1** | **1** | **1** |
| **200** | **0** | **0** | **0** | **0** | **0** | **0** | **0** | **0** | **0** | **0** |

| **ID** | **GPT_4o_1** | **GPT_4o_2** | **GPT_4o_3** | **GPT_4o_4** | **GPT_4o_5** | **GPT_4o_6** | **GPT_4o_7** | **GPT_4o_8** | **GPT_4o_9** | **GPT_4o_10** |
| --- | --- | --- | --- | --- | --- | --- | --- | --- | --- | --- |
| **1** | **1** | **1** | **1** | **1** | **1** | **1** | **1** | **1** | **1** | **1** |
| **2** | **1** | **1** | **1** | **1** | **1** | **1** | **1** | **1** | **1** | **1** |
| **3** | **0** | **0** | **0** | **0** | **0** | **0** | **0** | **0** | **0** | **0** |
| **4** | **1** | **1** | **1** | **1** | **1** | **1** | **1** | **1** | **1** | **1** |
| **5** | **1** | **1** | **1** | **1** | **1** | **1** | **1** | **1** | **1** | **1** |
| **6** | **1** | **1** | **1** | **1** | **1** | **1** | **1** | **1** | **1** | **1** |
| **7** | **1** | **1** | **1** | **1** | **1** | **1** | **1** | **1** | **1** | **1** |
| **8** | **1** | **1** | **1** | **1** | **1** | **1** | **1** | **1** | **1** | **1** |
| **9** | **1** | **1** | **1** | **1** | **1** | **1** | **1** | **1** | **1** | **1** |
| **10** | **1** | **1** | **1** | **1** | **1** | **1** | **1** | **1** | **1** | **1** |
| **11** | **1** | **1** | **1** | **1** | **1** | **1** | **1** | **1** | **1** | **1** |
| **12** | **1** | **1** | **1** | **1** | **1** | **1** | **1** | **1** | **1** | **1** |
| **13** | **1** | **1** | **1** | **1** | **1** | **1** | **1** | **1** | **1** | **1** |
| **14** | **1** | **1** | **1** | **1** | **1** | **1** | **1** | **1** | **1** | **1** |
| **15** | **1** | **1** | **1** | **1** | **1** | **1** | **1** | **1** | **1** | **1** |
| **16** | **0** | **0** | **0** | **0** | **0** | **0** | **0** | **0** | **0** | **0** |
| **17** | **1** | **1** | **1** | **1** | **1** | **1** | **1** | **1** | **1** | **1** |
| **18** | **1** | **1** | **1** | **1** | **1** | **1** | **1** | **1** | **1** | **1** |
| **19** | **0** | **0** | **0** | **0** | **0** | **0** | **0** | **0** | **0** | **0** |
| **20** | **1** | **1** | **1** | **1** | **1** | **1** | **1** | **1** | **1** | **1** |
| **21** | **1** | **1** | **1** | **1** | **1** | **1** | **1** | **1** | **1** | **1** |
| **22** | **0** | **0** | **0** | **0** | **0** | **0** | **0** | **0** | **0** | **0** |
| **23** | **1** | **1** | **1** | **1** | **1** | **1** | **1** | **1** | **1** | **1** |
| **24** | **0** | **0** | **0** | **0** | **0** | **0** | **0** | **0** | **0** | **0** |
| **25** | **1** | **1** | **1** | **1** | **1** | **1** | **1** | **1** | **1** | **1** |
| **26** | **1** | **0** | **1** | **0** | **1** | **1** | **1** | **1** | **1** | **1** |
| **27** | **1** | **1** | **1** | **1** | **1** | **1** | **1** | **1** | **1** | **1** |
| **28** | **1** | **1** | **1** | **1** | **1** | **1** | **1** | **1** | **1** | **1** |
| **29** | **1** | **1** | **1** | **1** | **1** | **1** | **1** | **1** | **1** | **1** |
| **30** | **1** | **1** | **1** | **1** | **1** | **1** | **1** | **1** | **1** | **1** |
| **31** | **1** | **1** | **1** | **1** | **1** | **1** | **1** | **1** | **1** | **1** |
| **32** | **0** | **0** | **0** | **0** | **0** | **0** | **0** | **0** | **0** | **0** |
| **33** | **1** | **1** | **1** | **1** | **1** | **1** | **1** | **1** | **1** | **1** |
| **34** | **1** | **1** | **1** | **1** | **1** | **1** | **1** | **1** | **1** | **1** |
| **35** | **1** | **1** | **1** | **1** | **1** | **1** | **1** | **1** | **1** | **1** |
| **36** | **1** | **1** | **1** | **1** | **1** | **1** | **1** | **1** | **1** | **1** |
| **37** | **0** | **0** | **0** | **0** | **0** | **0** | **0** | **0** | **0** | **0** |
| **38** | **0** | **0** | **0** | **0** | **0** | **0** | **0** | **0** | **0** | **0** |
| **39** | **0** | **0** | **0** | **0** | **0** | **0** | **0** | **0** | **0** | **0** |
| **40** | **1** | **1** | **1** | **1** | **1** | **1** | **1** | **1** | **1** | **1** |
| **41** | **1** | **1** | **1** | **1** | **1** | **1** | **1** | **1** | **1** | **1** |
| **42** | **1** | **1** | **1** | **1** | **1** | **1** | **1** | **1** | **1** | **1** |
| **43** | **0** | **0** | **0** | **0** | **0** | **0** | **0** | **0** | **0** | **0** |
| **44** | **1** | **1** | **1** | **1** | **1** | **1** | **1** | **1** | **1** | **1** |
| **45** | **1** | **1** | **1** | **1** | **1** | **1** | **1** | **1** | **1** | **1** |
| **46** | **1** | **1** | **1** | **1** | **1** | **1** | **1** | **1** | **1** | **1** |
| **47** | **0** | **0** | **0** | **0** | **0** | **0** | **0** | **0** | **0** | **0** |
| **48** | **1** | **1** | **1** | **1** | **1** | **1** | **1** | **1** | **1** | **1** |
| **49** | **1** | **1** | **1** | **1** | **1** | **1** | **1** | **1** | **1** | **1** |
| **50** | **1** | **1** | **1** | **1** | **1** | **1** | **1** | **1** | **1** | **1** |
| **51** | **0** | **0** | **1** | **1** | **1** | **1** | **0** | **0** | **1** | **1** |
| **52** | **1** | **1** | **1** | **1** | **1** | **1** | **1** | **1** | **1** | **1** |
| **53** | **1** | **1** | **1** | **1** | **1** | **1** | **1** | **1** | **1** | **1** |
| **54** | **1** | **1** | **1** | **1** | **1** | **0** | **1** | **1** | **0** | **1** |
| **55** | **1** | **1** | **1** | **1** | **1** | **1** | **1** | **1** | **1** | **1** |
| **56** | **1** | **1** | **1** | **1** | **1** | **1** | **1** | **1** | **1** | **1** |
| **57** | **1** | **1** | **1** | **1** | **1** | **1** | **1** | **1** | **1** | **1** |
| **58** | **1** | **1** | **1** | **1** | **1** | **1** | **1** | **1** | **1** | **1** |
| **59** | **1** | **1** | **1** | **1** | **1** | **1** | **1** | **1** | **1** | **1** |
| **60** | **0** | **0** | **0** | **0** | **0** | **0** | **0** | **0** | **0** | **0** |
| **61** | **1** | **1** | **1** | **1** | **1** | **1** | **1** | **1** | **1** | **1** |
| **62** | **1** | **1** | **1** | **1** | **1** | **1** | **1** | **1** | **1** | **1** |
| **63** | **1** | **1** | **1** | **1** | **1** | **1** | **1** | **1** | **1** | **1** |
| **64** | **1** | **1** | **1** | **1** | **1** | **1** | **1** | **1** | **1** | **1** |
| **65** | **1** | **1** | **1** | **1** | **1** | **1** | **1** | **1** | **1** | **1** |
| **66** | **1** | **1** | **1** | **1** | **1** | **1** | **1** | **1** | **1** | **1** |
| **67** | **1** | **1** | **1** | **1** | **1** | **1** | **1** | **1** | **1** | **1** |
| **68** | **0** | **0** | **0** | **0** | **0** | **0** | **0** | **0** | **0** | **0** |
| **69** | **0** | **0** | **0** | **0** | **0** | **0** | **0** | **0** | **0** | **0** |
| **70** | **1** | **1** | **1** | **1** | **1** | **1** | **1** | **1** | **1** | **1** |
| **71** | **1** | **1** | **1** | **1** | **1** | **1** | **1** | **1** | **1** | **1** |
| **72** | **1** | **1** | **1** | **1** | **1** | **1** | **1** | **1** | **1** | **1** |
| **73** | **1** | **1** | **1** | **1** | **1** | **1** | **1** | **1** | **1** | **1** |
| **74** | **1** | **1** | **1** | **1** | **1** | **1** | **1** | **1** | **1** | **1** |
| **75** | **1** | **1** | **1** | **1** | **1** | **1** | **1** | **1** | **1** | **1** |
| **76** | **1** | **1** | **1** | **1** | **1** | **1** | **1** | **1** | **1** | **1** |
| **77** | **1** | **1** | **1** | **1** | **1** | **1** | **1** | **1** | **1** | **1** |
| **78** | **1** | **1** | **1** | **1** | **1** | **1** | **1** | **1** | **1** | **1** |
| **79** | **1** | **1** | **1** | **1** | **1** | **1** | **1** | **1** | **1** | **1** |
| **80** | **0** | **0** | **0** | **0** | **0** | **0** | **0** | **0** | **0** | **0** |
| **81** | **0** | **0** | **0** | **0** | **0** | **0** | **0** | **0** | **0** | **0** |
| **82** | **0** | **0** | **0** | **0** | **0** | **0** | **0** | **0** | **0** | **0** |
| **83** | **1** | **1** | **1** | **1** | **1** | **1** | **1** | **1** | **1** | **1** |
| **84** | **1** | **1** | **1** | **1** | **1** | **1** | **1** | **1** | **1** | **1** |
| **85** | **0** | **0** | **0** | **0** | **0** | **0** | **0** | **0** | **0** | **0** |
| **86** | **1** | **1** | **1** | **1** | **1** | **1** | **1** | **1** | **1** | **1** |
| **87** | **1** | **1** | **1** | **1** | **1** | **1** | **1** | **1** | **1** | **1** |
| **88** | **1** | **1** | **0** | **1** | **1** | **0** | **0** | **1** | **0** | **0** |
| **89** | **1** | **1** | **1** | **1** | **1** | **1** | **1** | **1** | **1** | **1** |
| **90** | **1** | **1** | **1** | **1** | **1** | **1** | **1** | **1** | **1** | **1** |
| **91** | **1** | **1** | **1** | **1** | **1** | **1** | **1** | **1** | **1** | **1** |
| **92** | **1** | **1** | **1** | **1** | **1** | **1** | **1** | **1** | **1** | **1** |
| **93** | **1** | **1** | **1** | **1** | **1** | **1** | **1** | **1** | **1** | **1** |
| **94** | **1** | **1** | **1** | **1** | **1** | **1** | **1** | **1** | **1** | **1** |
| **95** | **1** | **1** | **1** | **1** | **1** | **1** | **1** | **1** | **1** | **1** |
| **96** | **1** | **1** | **1** | **1** | **1** | **1** | **1** | **1** | **1** | **1** |
| **97** | **1** | **1** | **1** | **1** | **1** | **1** | **1** | **1** | **1** | **1** |
| **98** | **0** | **0** | **0** | **0** | **0** | **0** | **0** | **0** | **0** | **0** |
| **99** | **1** | **1** | **1** | **1** | **1** | **1** | **1** | **1** | **1** | **1** |
| **100** | **1** | **1** | **1** | **1** | **1** | **1** | **1** | **1** | **1** | **1** |
| **101** | **1** | **0** | **0** | **1** | **0** | **0** | **0** | **0** | **0** | **0** |
| **102** | **1** | **1** | **1** | **1** | **1** | **1** | **1** | **1** | **1** | **1** |
| **103** | **1** | **1** | **1** | **1** | **1** | **1** | **1** | **1** | **1** | **1** |
| **104** | **0** | **0** | **0** | **0** | **0** | **0** | **0** | **0** | **0** | **0** |
| **105** | **1** | **1** | **1** | **1** | **1** | **1** | **1** | **1** | **1** | **1** |
| **106** | **0** | **0** | **0** | **0** | **0** | **0** | **0** | **0** | **0** | **0** |
| **107** | **1** | **1** | **1** | **1** | **1** | **1** | **1** | **1** | **1** | **1** |
| **108** | **1** | **1** | **1** | **1** | **1** | **1** | **1** | **1** | **1** | **1** |
| **109** | **1** | **1** | **1** | **1** | **1** | **1** | **1** | **1** | **1** | **1** |
| **110** | **0** | **0** | **0** | **0** | **0** | **0** | **0** | **0** | **0** | **0** |
| **111** | **1** | **1** | **1** | **1** | **1** | **1** | **1** | **1** | **1** | **1** |
| **112** | **0** | **0** | **1** | **0** | **1** | **1** | **1** | **1** | **0** | **1** |
| **113** | **1** | **1** | **1** | **1** | **1** | **1** | **1** | **1** | **1** | **1** |
| **114** | **1** | **1** | **1** | **1** | **1** | **1** | **1** | **1** | **1** | **1** |
| **115** | **0** | **0** | **0** | **0** | **0** | **0** | **0** | **0** | **0** | **0** |
| **116** | **1** | **1** | **1** | **1** | **1** | **1** | **1** | **1** | **1** | **1** |
| **117** | **0** | **0** | **0** | **0** | **0** | **0** | **0** | **0** | **0** | **0** |
| **118** | **1** | **1** | **1** | **1** | **1** | **1** | **1** | **1** | **1** | **1** |
| **119** | **1** | **1** | **1** | **1** | **1** | **1** | **1** | **1** | **1** | **1** |
| **120** | **1** | **1** | **1** | **1** | **1** | **1** | **1** | **1** | **1** | **1** |
| **121** | **1** | **1** | **1** | **1** | **1** | **1** | **1** | **1** | **1** | **1** |
| **122** | **1** | **1** | **1** | **1** | **1** | **1** | **1** | **1** | **1** | **1** |
| **123** | **0** | **0** | **0** | **0** | **0** | **0** | **0** | **0** | **0** | **0** |
| **124** | **1** | **1** | **1** | **1** | **1** | **1** | **1** | **1** | **1** | **1** |
| **125** | **1** | **1** | **1** | **1** | **1** | **1** | **1** | **1** | **1** | **1** |
| **126** | **0** | **0** | **0** | **0** | **0** | **0** | **0** | **0** | **0** | **0** |
| **127** | **1** | **1** | **1** | **1** | **1** | **1** | **1** | **1** | **1** | **1** |
| **128** | **1** | **1** | **1** | **1** | **1** | **1** | **1** | **1** | **1** | **1** |
| **129** | **0** | **0** | **0** | **0** | **0** | **0** | **0** | **0** | **0** | **0** |
| **130** | **1** | **1** | **1** | **1** | **1** | **1** | **1** | **1** | **1** | **1** |
| **131** | **0** | **0** | **0** | **0** | **0** | **0** | **0** | **0** | **0** | **0** |
| **132** | **1** | **1** | **1** | **1** | **1** | **1** | **1** | **1** | **1** | **1** |
| **133** | **1** | **1** | **1** | **1** | **1** | **1** | **1** | **1** | **1** | **1** |
| **134** | **1** | **1** | **1** | **1** | **1** | **1** | **1** | **1** | **1** | **1** |
| **135** | **1** | **1** | **1** | **1** | **1** | **1** | **1** | **1** | **1** | **1** |
| **136** | **1** | **1** | **1** | **1** | **1** | **1** | **1** | **1** | **1** | **1** |
| **137** | **1** | **1** | **1** | **1** | **1** | **1** | **1** | **1** | **1** | **1** |
| **138** | **1** | **1** | **1** | **1** | **1** | **1** | **1** | **1** | **1** | **1** |
| **139** | **0** | **0** | **0** | **0** | **0** | **0** | **0** | **0** | **0** | **0** |
| **140** | **0** | **1** | **1** | **0** | **0** | **0** | **1** | **1** | **0** | **0** |
| **141** | **1** | **1** | **1** | **1** | **1** | **1** | **1** | **1** | **1** | **1** |
| **142** | **1** | **1** | **1** | **1** | **1** | **1** | **1** | **1** | **1** | **1** |
| **143** | **1** | **1** | **1** | **1** | **1** | **1** | **1** | **1** | **1** | **1** |
| **144** | **1** | **1** | **1** | **1** | **1** | **1** | **1** | **1** | **1** | **1** |
| **145** | **1** | **1** | **1** | **1** | **1** | **1** | **1** | **1** | **1** | **1** |
| **146** | **0** | **0** | **0** | **0** | **0** | **0** | **0** | **0** | **0** | **0** |
| **147** | **1** | **1** | **1** | **1** | **1** | **1** | **1** | **1** | **1** | **1** |
| **148** | **1** | **1** | **1** | **1** | **1** | **1** | **1** | **1** | **1** | **1** |
| **149** | **1** | **1** | **1** | **1** | **1** | **1** | **1** | **1** | **1** | **1** |
| **150** | **1** | **1** | **1** | **1** | **1** | **1** | **1** | **1** | **1** | **1** |
| **151** | **1** | **1** | **1** | **1** | **1** | **1** | **1** | **1** | **1** | **1** |
| **152** | **0** | **0** | **0** | **0** | **0** | **0** | **0** | **0** | **0** | **0** |
| **153** | **0** | **1** | **1** | **0** | **0** | **1** | **0** | **0** | **0** | **0** |
| **154** | **1** | **1** | **1** | **1** | **1** | **1** | **1** | **1** | **1** | **1** |
| **155** | **1** | **1** | **1** | **1** | **1** | **1** | **1** | **1** | **1** | **1** |
| **156** | **1** | **1** | **1** | **1** | **1** | **1** | **1** | **1** | **1** | **1** |
| **157** | **1** | **1** | **1** | **1** | **1** | **1** | **1** | **1** | **1** | **1** |
| **158** | **1** | **1** | **1** | **1** | **1** | **1** | **1** | **1** | **1** | **1** |
| **159** | **1** | **1** | **1** | **1** | **1** | **1** | **1** | **1** | **1** | **1** |
| **160** | **1** | **1** | **1** | **1** | **1** | **1** | **1** | **1** | **1** | **1** |
| **161** | **1** | **1** | **1** | **1** | **1** | **1** | **1** | **1** | **1** | **1** |
| **162** | **1** | **1** | **1** | **1** | **1** | **1** | **1** | **1** | **1** | **1** |
| **163** | **1** | **1** | **1** | **1** | **1** | **1** | **1** | **1** | **1** | **1** |
| **164** | **1** | **1** | **1** | **1** | **1** | **1** | **1** | **1** | **1** | **1** |
| **165** | **1** | **1** | **1** | **1** | **1** | **1** | **1** | **1** | **1** | **1** |
| **166** | **1** | **1** | **1** | **1** | **1** | **1** | **1** | **1** | **1** | **1** |
| **167** | **1** | **1** | **1** | **1** | **1** | **1** | **1** | **1** | **1** | **1** |
| **168** | **1** | **1** | **1** | **1** | **1** | **1** | **1** | **1** | **1** | **1** |
| **169** | **1** | **1** | **1** | **1** | **1** | **1** | **1** | **1** | **1** | **1** |
| **170** | **1** | **1** | **1** | **1** | **1** | **1** | **1** | **1** | **1** | **1** |
| **171** | **0** | **0** | **0** | **0** | **0** | **0** | **0** | **0** | **0** | **0** |
| **172** | **0** | **0** | **1** | **1** | **1** | **1** | **1** | **1** | **1** | **1** |
| **173** | **1** | **1** | **1** | **1** | **1** | **1** | **1** | **1** | **1** | **1** |
| **174** | **1** | **1** | **1** | **1** | **1** | **1** | **1** | **1** | **1** | **1** |
| **175** | **1** | **1** | **1** | **1** | **1** | **1** | **1** | **1** | **1** | **1** |
| **176** | **1** | **1** | **1** | **1** | **1** | **1** | **1** | **1** | **1** | **1** |
| **177** | **1** | **1** | **1** | **1** | **1** | **1** | **1** | **1** | **1** | **1** |
| **178** | **1** | **1** | **1** | **1** | **1** | **1** | **1** | **1** | **1** | **1** |
| **179** | **0** | **0** | **0** | **0** | **0** | **0** | **0** | **0** | **0** | **0** |
| **180** | **0** | **0** | **0** | **0** | **0** | **0** | **0** | **0** | **0** | **0** |
| **181** | **1** | **1** | **1** | **1** | **1** | **1** | **1** | **1** | **1** | **1** |
| **182** | **1** | **1** | **1** | **1** | **1** | **1** | **1** | **1** | **1** | **1** |
| **183** | **1** | **1** | **0** | **1** | **0** | **0** | **1** | **1** | **1** | **1** |
| **184** | **1** | **1** | **1** | **1** | **1** | **1** | **1** | **1** | **1** | **1** |
| **185** | **0** | **0** | **0** | **0** | **0** | **0** | **0** | **0** | **0** | **0** |
| **186** | **1** | **1** | **1** | **1** | **1** | **1** | **1** | **1** | **1** | **1** |
| **187** | **1** | **0** | **0** | **1** | **0** | **0** | **0** | **0** | **0** | **1** |
| **188** | **1** | **0** | **0** | **1** | **0** | **0** | **0** | **0** | **0** | **0** |
| **189** | **0** | **1** | **0** | **1** | **1** | **1** | **0** | **1** | **0** | **0** |
| **190** | **1** | **1** | **1** | **1** | **1** | **1** | **1** | **1** | **1** | **1** |
| **191** | **1** | **1** | **1** | **1** | **1** | **1** | **1** | **1** | **1** | **1** |
| **192** | **0** | **0** | **0** | **0** | **0** | **0** | **0** | **0** | **0** | **0** |
| **193** | **1** | **1** | **1** | **1** | **1** | **1** | **1** | **1** | **1** | **1** |
| **194** | **1** | **1** | **0** | **1** | **1** | **1** | **1** | **1** | **1** | **1** |
| **195** | **1** | **1** | **1** | **1** | **1** | **1** | **1** | **1** | **1** | **1** |
| **196** | **1** | **0** | **1** | **1** | **0** | **0** | **1** | **0** | **1** | **1** |
| **197** | **1** | **1** | **1** | **1** | **1** | **1** | **1** | **1** | **1** | **1** |
| **198** | **0** | **0** | **0** | **0** | **0** | **0** | **0** | **0** | **0** | **0** |
| **199** | **1** | **1** | **1** | **1** | **1** | **1** | **1** | **1** | **1** | **1** |
| **200** | **0** | **0** | **0** | **0** | **0** | **0** | **0** | **0** | **0** | **0** |

| **ID** | **UroBot_4o_1** | **UroBot_4o_2** | **UroBot_4o_3** | **UroBot_4o_4** | **UroBot_4o_5** | **UroBot_4o_6** | **UroBot_4o_7** | **UroBot_4o_8** | **UroBot_4o_9** | **UroBot_4o_10** |
| --- | --- | --- | --- | --- | --- | --- | --- | --- | --- | --- |
| **1** | **1** | **1** | **1** | **1** | **1** | **1** | **1** | **1** | **1** | **1** |
| **2** | **1** | **1** | **1** | **1** | **1** | **1** | **1** | **1** | **1** | **1** |
| **3** | **1** | **1** | **1** | **1** | **1** | **1** | **1** | **1** | **1** | **1** |
| **4** | **1** | **1** | **1** | **1** | **1** | **1** | **1** | **1** | **1** | **1** |
| **5** | **1** | **1** | **1** | **1** | **1** | **1** | **1** | **1** | **1** | **1** |
| **6** | **1** | **1** | **1** | **1** | **1** | **1** | **1** | **1** | **1** | **1** |
| **7** | **1** | **1** | **1** | **1** | **1** | **1** | **1** | **1** | **1** | **1** |
| **8** | **1** | **1** | **1** | **1** | **1** | **1** | **1** | **1** | **1** | **1** |
| **9** | **1** | **1** | **1** | **1** | **1** | **1** | **1** | **1** | **1** | **1** |
| **10** | **1** | **1** | **1** | **1** | **1** | **1** | **1** | **1** | **1** | **1** |
| **11** | **1** | **1** | **1** | **1** | **1** | **1** | **1** | **1** | **1** | **1** |
| **12** | **1** | **1** | **1** | **1** | **1** | **1** | **1** | **1** | **1** | **1** |
| **13** | **1** | **1** | **1** | **1** | **1** | **1** | **1** | **1** | **1** | **1** |
| **14** | **1** | **1** | **1** | **1** | **1** | **1** | **1** | **1** | **1** | **1** |
| **15** | **1** | **1** | **1** | **1** | **1** | **1** | **1** | **1** | **1** | **1** |
| **16** | **1** | **1** | **1** | **1** | **1** | **1** | **1** | **1** | **1** | **1** |
| **17** | **1** | **1** | **1** | **1** | **1** | **1** | **1** | **1** | **1** | **1** |
| **18** | **1** | **1** | **1** | **1** | **1** | **1** | **1** | **1** | **1** | **1** |
| **19** | **1** | **1** | **1** | **1** | **1** | **1** | **1** | **1** | **1** | **1** |
| **20** | **1** | **1** | **1** | **1** | **1** | **1** | **1** | **1** | **1** | **1** |
| **21** | **1** | **1** | **1** | **1** | **1** | **1** | **1** | **1** | **1** | **1** |
| **22** | **0** | **0** | **0** | **0** | **0** | **0** | **0** | **0** | **0** | **0** |
| **23** | **1** | **1** | **1** | **1** | **1** | **1** | **1** | **1** | **1** | **1** |
| **24** | **1** | **0** | **0** | **0** | **0** | **0** | **0** | **0** | **0** | **0** |
| **25** | **1** | **1** | **1** | **1** | **1** | **1** | **1** | **1** | **1** | **1** |
| **26** | **1** | **1** | **1** | **1** | **1** | **1** | **1** | **1** | **1** | **1** |
| **27** | **1** | **1** | **1** | **1** | **1** | **1** | **1** | **1** | **1** | **1** |
| **28** | **1** | **1** | **1** | **1** | **1** | **1** | **1** | **1** | **1** | **1** |
| **29** | **1** | **1** | **1** | **1** | **1** | **1** | **1** | **1** | **1** | **1** |
| **30** | **1** | **1** | **1** | **1** | **1** | **1** | **1** | **1** | **1** | **1** |
| **31** | **1** | **1** | **1** | **1** | **1** | **1** | **1** | **1** | **1** | **1** |
| **32** | **1** | **1** | **1** | **1** | **1** | **1** | **1** | **1** | **1** | **1** |
| **33** | **1** | **1** | **1** | **1** | **1** | **1** | **1** | **1** | **1** | **1** |
| **34** | **1** | **1** | **1** | **1** | **1** | **1** | **1** | **1** | **1** | **1** |
| **35** | **1** | **1** | **1** | **1** | **1** | **1** | **1** | **1** | **1** | **1** |
| **36** | **1** | **1** | **1** | **1** | **1** | **1** | **1** | **1** | **1** | **1** |
| **37** | **0** | **0** | **0** | **0** | **0** | **0** | **0** | **0** | **0** | **0** |
| **38** | **0** | **0** | **0** | **0** | **0** | **0** | **0** | **0** | **0** | **0** |
| **39** | **1** | **1** | **1** | **1** | **1** | **1** | **1** | **1** | **1** | **1** |
| **40** | **1** | **1** | **1** | **1** | **1** | **1** | **1** | **1** | **1** | **1** |
| **41** | **0** | **0** | **0** | **0** | **0** | **0** | **0** | **0** | **0** | **0** |
| **42** | **1** | **1** | **1** | **1** | **1** | **1** | **1** | **1** | **1** | **1** |
| **43** | **1** | **1** | **1** | **1** | **1** | **1** | **1** | **1** | **1** | **1** |
| **44** | **1** | **1** | **1** | **1** | **1** | **1** | **1** | **1** | **1** | **1** |
| **45** | **1** | **1** | **1** | **1** | **1** | **1** | **1** | **1** | **1** | **1** |
| **46** | **1** | **1** | **1** | **1** | **1** | **1** | **1** | **1** | **1** | **1** |
| **47** | **1** | **1** | **1** | **1** | **1** | **1** | **1** | **1** | **1** | **1** |
| **48** | **1** | **1** | **1** | **1** | **1** | **1** | **1** | **1** | **1** | **1** |
| **49** | **1** | **1** | **1** | **1** | **1** | **1** | **1** | **1** | **1** | **1** |
| **50** | **1** | **1** | **1** | **1** | **1** | **1** | **1** | **1** | **1** | **1** |
| **51** | **1** | **1** | **1** | **1** | **1** | **1** | **1** | **1** | **1** | **1** |
| **52** | **1** | **1** | **1** | **1** | **1** | **1** | **1** | **1** | **1** | **1** |
| **53** | **1** | **1** | **1** | **1** | **1** | **1** | **1** | **1** | **1** | **1** |
| **54** | **1** | **1** | **1** | **1** | **1** | **1** | **1** | **1** | **1** | **1** |
| **55** | **1** | **1** | **1** | **1** | **1** | **1** | **1** | **1** | **1** | **1** |
| **56** | **1** | **1** | **1** | **1** | **1** | **1** | **1** | **1** | **1** | **1** |
| **57** | **1** | **1** | **1** | **1** | **1** | **1** | **1** | **1** | **1** | **1** |
| **58** | **1** | **1** | **1** | **1** | **1** | **1** | **1** | **1** | **1** | **1** |
| **59** | **1** | **1** | **1** | **1** | **1** | **1** | **1** | **1** | **1** | **1** |
| **60** | **1** | **1** | **1** | **1** | **1** | **1** | **1** | **1** | **1** | **1** |
| **61** | **0** | **0** | **0** | **0** | **0** | **0** | **0** | **0** | **0** | **0** |
| **62** | **1** | **1** | **1** | **1** | **0** | **1** | **1** | **1** | **1** | **0** |
| **63** | **1** | **1** | **1** | **1** | **1** | **1** | **1** | **1** | **1** | **1** |
| **64** | **1** | **1** | **1** | **1** | **1** | **1** | **1** | **1** | **1** | **1** |
| **65** | **1** | **1** | **1** | **1** | **1** | **1** | **1** | **1** | **1** | **1** |
| **66** | **1** | **1** | **1** | **1** | **1** | **1** | **1** | **1** | **1** | **1** |
| **67** | **1** | **1** | **1** | **1** | **1** | **1** | **1** | **1** | **1** | **1** |
| **68** | **1** | **1** | **1** | **1** | **1** | **1** | **1** | **1** | **1** | **1** |
| **69** | **1** | **1** | **1** | **1** | **1** | **1** | **1** | **1** | **1** | **1** |
| **70** | **1** | **1** | **1** | **1** | **1** | **1** | **1** | **1** | **1** | **1** |
| **71** | **1** | **1** | **1** | **1** | **1** | **1** | **1** | **1** | **1** | **1** |
| **72** | **1** | **1** | **1** | **1** | **1** | **1** | **1** | **1** | **1** | **1** |
| **73** | **1** | **1** | **1** | **1** | **1** | **1** | **1** | **1** | **1** | **1** |
| **74** | **1** | **1** | **1** | **1** | **1** | **1** | **1** | **1** | **1** | **1** |
| **75** | **1** | **1** | **1** | **1** | **1** | **1** | **1** | **1** | **1** | **1** |
| **76** | **1** | **1** | **1** | **1** | **1** | **1** | **1** | **1** | **1** | **1** |
| **77** | **1** | **1** | **1** | **1** | **1** | **1** | **1** | **1** | **1** | **1** |
| **78** | **1** | **1** | **1** | **1** | **1** | **1** | **1** | **1** | **1** | **1** |
| **79** | **1** | **1** | **1** | **1** | **1** | **1** | **1** | **1** | **1** | **1** |
| **80** | **0** | **0** | **0** | **0** | **0** | **0** | **0** | **0** | **0** | **0** |
| **81** | **1** | **1** | **1** | **1** | **1** | **1** | **1** | **1** | **1** | **1** |
| **82** | **0** | **0** | **0** | **0** | **0** | **0** | **0** | **0** | **0** | **0** |
| **83** | **1** | **1** | **1** | **1** | **1** | **1** | **1** | **1** | **1** | **1** |
| **84** | **0** | **0** | **0** | **0** | **0** | **0** | **0** | **0** | **0** | **0** |
| **85** | **0** | **0** | **0** | **0** | **0** | **0** | **0** | **0** | **0** | **0** |
| **86** | **1** | **1** | **1** | **1** | **1** | **1** | **1** | **1** | **1** | **1** |
| **87** | **1** | **1** | **1** | **1** | **1** | **1** | **1** | **1** | **1** | **1** |
| **88** | **1** | **1** | **1** | **1** | **1** | **1** | **1** | **1** | **1** | **1** |
| **89** | **1** | **1** | **1** | **1** | **1** | **1** | **1** | **1** | **1** | **1** |
| **90** | **1** | **1** | **1** | **1** | **1** | **1** | **1** | **1** | **1** | **1** |
| **91** | **1** | **1** | **1** | **1** | **1** | **1** | **1** | **1** | **1** | **1** |
| **92** | **1** | **1** | **1** | **1** | **1** | **1** | **1** | **1** | **1** | **1** |
| **93** | **1** | **1** | **1** | **1** | **1** | **1** | **1** | **1** | **1** | **1** |
| **94** | **1** | **1** | **1** | **1** | **1** | **1** | **1** | **1** | **1** | **1** |
| **95** | **1** | **1** | **1** | **1** | **1** | **1** | **1** | **1** | **1** | **1** |
| **96** | **1** | **1** | **1** | **1** | **1** | **1** | **1** | **1** | **1** | **1** |
| **97** | **1** | **1** | **1** | **1** | **1** | **1** | **1** | **1** | **1** | **1** |
| **98** | **1** | **1** | **1** | **1** | **1** | **1** | **1** | **1** | **1** | **1** |
| **99** | **1** | **1** | **1** | **1** | **1** | **1** | **1** | **1** | **1** | **1** |
| **100** | **1** | **1** | **1** | **1** | **1** | **1** | **1** | **1** | **1** | **1** |
| **101** | **1** | **1** | **1** | **1** | **1** | **1** | **1** | **1** | **1** | **1** |
| **102** | **1** | **1** | **1** | **1** | **1** | **1** | **1** | **1** | **1** | **1** |
| **103** | **1** | **1** | **1** | **1** | **1** | **1** | **1** | **1** | **1** | **1** |
| **104** | **0** | **0** | **0** | **0** | **0** | **0** | **0** | **0** | **0** | **0** |
| **105** | **1** | **1** | **1** | **1** | **1** | **1** | **1** | **1** | **1** | **1** |
| **106** | **1** | **1** | **1** | **1** | **1** | **1** | **1** | **1** | **1** | **1** |
| **107** | **1** | **1** | **1** | **1** | **1** | **1** | **1** | **1** | **1** | **1** |
| **108** | **1** | **1** | **1** | **1** | **1** | **1** | **1** | **1** | **1** | **1** |
| **109** | **1** | **1** | **1** | **1** | **1** | **1** | **1** | **1** | **1** | **1** |
| **110** | **1** | **1** | **1** | **1** | **1** | **1** | **1** | **1** | **1** | **1** |
| **111** | **1** | **1** | **1** | **1** | **1** | **1** | **1** | **1** | **1** | **1** |
| **112** | **0** | **0** | **0** | **0** | **0** | **0** | **0** | **0** | **0** | **0** |
| **113** | **1** | **1** | **1** | **1** | **1** | **1** | **1** | **1** | **1** | **1** |
| **114** | **1** | **1** | **1** | **1** | **1** | **1** | **1** | **1** | **1** | **1** |
| **115** | **0** | **0** | **0** | **0** | **0** | **0** | **0** | **0** | **0** | **0** |
| **116** | **1** | **1** | **1** | **1** | **1** | **1** | **1** | **1** | **1** | **1** |
| **117** | **0** | **0** | **0** | **0** | **0** | **0** | **0** | **0** | **0** | **0** |
| **118** | **1** | **1** | **1** | **1** | **1** | **1** | **1** | **1** | **1** | **1** |
| **119** | **1** | **1** | **1** | **1** | **1** | **1** | **1** | **1** | **1** | **1** |
| **120** | **1** | **1** | **1** | **1** | **1** | **1** | **1** | **1** | **1** | **1** |
| **121** | **1** | **1** | **1** | **1** | **1** | **1** | **1** | **1** | **1** | **1** |
| **122** | **1** | **1** | **1** | **1** | **1** | **1** | **1** | **1** | **1** | **1** |
| **123** | **0** | **0** | **0** | **0** | **0** | **0** | **0** | **0** | **0** | **0** |
| **124** | **1** | **1** | **1** | **1** | **1** | **1** | **1** | **1** | **1** | **1** |
| **125** | **1** | **1** | **1** | **1** | **1** | **1** | **1** | **1** | **1** | **1** |
| **126** | **1** | **1** | **1** | **1** | **1** | **1** | **1** | **1** | **1** | **1** |
| **127** | **1** | **1** | **1** | **1** | **1** | **1** | **1** | **1** | **1** | **1** |
| **128** | **1** | **1** | **1** | **1** | **1** | **1** | **1** | **1** | **1** | **1** |
| **129** | **1** | **1** | **1** | **1** | **1** | **1** | **1** | **1** | **1** | **1** |
| **130** | **1** | **1** | **1** | **1** | **1** | **1** | **1** | **1** | **1** | **1** |
| **131** | **1** | **1** | **1** | **1** | **1** | **1** | **1** | **1** | **1** | **1** |
| **132** | **1** | **1** | **1** | **1** | **1** | **1** | **1** | **1** | **1** | **1** |
| **133** | **1** | **1** | **1** | **1** | **1** | **1** | **1** | **1** | **1** | **1** |
| **134** | **1** | **1** | **1** | **1** | **1** | **1** | **1** | **1** | **1** | **1** |
| **135** | **1** | **1** | **1** | **1** | **1** | **1** | **1** | **1** | **1** | **1** |
| **136** | **1** | **1** | **1** | **1** | **1** | **1** | **1** | **1** | **1** | **1** |
| **137** | **1** | **1** | **1** | **1** | **1** | **1** | **1** | **1** | **1** | **1** |
| **138** | **1** | **1** | **1** | **1** | **1** | **1** | **1** | **1** | **1** | **1** |
| **139** | **1** | **0** | **0** | **0** | **0** | **0** | **0** | **0** | **0** | **1** |
| **140** | **1** | **1** | **1** | **1** | **1** | **1** | **1** | **1** | **1** | **1** |
| **141** | **1** | **1** | **1** | **1** | **1** | **1** | **1** | **1** | **1** | **1** |
| **142** | **1** | **1** | **1** | **1** | **1** | **1** | **1** | **1** | **1** | **1** |
| **143** | **1** | **1** | **1** | **1** | **1** | **1** | **1** | **1** | **1** | **1** |
| **144** | **1** | **1** | **1** | **1** | **1** | **1** | **1** | **1** | **1** | **1** |
| **145** | **1** | **1** | **1** | **1** | **1** | **1** | **1** | **1** | **1** | **1** |
| **146** | **1** | **1** | **1** | **1** | **1** | **1** | **1** | **1** | **1** | **1** |
| **147** | **1** | **1** | **1** | **1** | **1** | **1** | **1** | **1** | **1** | **1** |
| **148** | **1** | **1** | **1** | **1** | **1** | **1** | **1** | **0** | **1** | **0** |
| **149** | **1** | **1** | **1** | **1** | **1** | **1** | **1** | **1** | **1** | **1** |
| **150** | **1** | **1** | **1** | **1** | **1** | **1** | **1** | **1** | **1** | **1** |
| **151** | **1** | **1** | **1** | **1** | **1** | **1** | **1** | **1** | **1** | **1** |
| **152** | **1** | **1** | **1** | **1** | **1** | **1** | **1** | **1** | **1** | **1** |
| **153** | **0** | **0** | **0** | **0** | **0** | **0** | **0** | **0** | **0** | **0** |
| **154** | **1** | **1** | **1** | **1** | **1** | **1** | **1** | **1** | **1** | **1** |
| **155** | **1** | **1** | **1** | **1** | **1** | **1** | **1** | **1** | **1** | **1** |
| **156** | **1** | **1** | **1** | **1** | **1** | **1** | **1** | **1** | **1** | **1** |
| **157** | **1** | **1** | **1** | **1** | **1** | **1** | **1** | **1** | **1** | **1** |
| **158** | **1** | **1** | **1** | **1** | **1** | **1** | **1** | **1** | **1** | **1** |
| **159** | **1** | **1** | **1** | **1** | **1** | **1** | **1** | **1** | **1** | **1** |
| **160** | **1** | **1** | **1** | **1** | **1** | **1** | **1** | **1** | **1** | **1** |
| **161** | **1** | **1** | **1** | **1** | **1** | **1** | **1** | **1** | **1** | **1** |
| **162** | **1** | **1** | **1** | **1** | **1** | **1** | **1** | **1** | **1** | **1** |
| **163** | **1** | **1** | **1** | **1** | **1** | **1** | **1** | **1** | **1** | **1** |
| **164** | **1** | **1** | **1** | **1** | **1** | **1** | **1** | **1** | **1** | **1** |
| **165** | **1** | **1** | **1** | **1** | **1** | **1** | **1** | **1** | **1** | **1** |
| **166** | **1** | **1** | **1** | **1** | **1** | **1** | **1** | **1** | **1** | **1** |
| **167** | **1** | **1** | **1** | **1** | **1** | **1** | **1** | **1** | **1** | **1** |
| **168** | **1** | **1** | **1** | **1** | **1** | **1** | **1** | **1** | **1** | **1** |
| **169** | **1** | **1** | **1** | **1** | **1** | **1** | **1** | **1** | **1** | **1** |
| **170** | **1** | **1** | **1** | **1** | **1** | **1** | **1** | **1** | **1** | **1** |
| **171** | **0** | **0** | **0** | **0** | **0** | **0** | **0** | **0** | **0** | **0** |
| **172** | **0** | **0** | **0** | **0** | **0** | **0** | **0** | **0** | **0** | **0** |
| **173** | **1** | **1** | **1** | **1** | **1** | **1** | **1** | **1** | **1** | **1** |
| **174** | **1** | **1** | **1** | **1** | **1** | **1** | **1** | **1** | **1** | **1** |
| **175** | **1** | **1** | **1** | **1** | **1** | **1** | **1** | **1** | **1** | **1** |
| **176** | **1** | **1** | **1** | **1** | **1** | **1** | **1** | **1** | **1** | **1** |
| **177** | **1** | **1** | **1** | **1** | **1** | **1** | **1** | **1** | **1** | **1** |
| **178** | **1** | **1** | **1** | **1** | **1** | **1** | **1** | **1** | **1** | **1** |
| **179** | **0** | **0** | **0** | **0** | **0** | **0** | **0** | **0** | **0** | **0** |
| **180** | **1** | **1** | **1** | **1** | **1** | **1** | **1** | **1** | **1** | **1** |
| **181** | **1** | **1** | **1** | **1** | **1** | **1** | **1** | **1** | **1** | **1** |
| **182** | **1** | **1** | **1** | **1** | **1** | **1** | **1** | **1** | **1** | **1** |
| **183** | **1** | **1** | **1** | **1** | **1** | **1** | **1** | **1** | **1** | **1** |
| **184** | **1** | **1** | **1** | **1** | **1** | **1** | **1** | **1** | **1** | **1** |
| **185** | **1** | **1** | **1** | **1** | **1** | **1** | **1** | **1** | **1** | **1** |
| **186** | **1** | **1** | **1** | **1** | **1** | **1** | **1** | **1** | **1** | **1** |
| **187** | **1** | **1** | **1** | **1** | **1** | **1** | **1** | **1** | **1** | **1** |
| **188** | **1** | **1** | **1** | **1** | **1** | **1** | **1** | **1** | **1** | **1** |
| **189** | **0** | **0** | **0** | **0** | **0** | **1** | **0** | **0** | **0** | **0** |
| **190** | **1** | **1** | **1** | **1** | **1** | **1** | **1** | **1** | **1** | **1** |
| **191** | **1** | **1** | **1** | **1** | **1** | **1** | **1** | **1** | **1** | **1** |
| **192** | **0** | **0** | **0** | **0** | **0** | **0** | **0** | **0** | **0** | **0** |
| **193** | **0** | **0** | **0** | **0** | **0** | **0** | **0** | **0** | **0** | **0** |
| **194** | **1** | **1** | **1** | **1** | **1** | **1** | **1** | **1** | **1** | **1** |
| **195** | **1** | **1** | **1** | **1** | **1** | **1** | **1** | **1** | **1** | **1** |
| **196** | **1** | **1** | **1** | **1** | **1** | **1** | **1** | **1** | **1** | **1** |
| **197** | **1** | **1** | **1** | **1** | **1** | **1** | **1** | **1** | **1** | **1** |
| **198** | **1** | **1** | **1** | **1** | **1** | **1** | **1** | **1** | **1** | **1** |
| **199** | **1** | **1** | **1** | **1** | **1** | **1** | **1** | **1** | **1** | **1** |
| **200** | **0** | **1** | **1** | **0** | **1** | **1** | **1** | **1** | **0** | **1** |

**References:**

- MCQ ISA booklet 2021-2022 of the European Board of Urology, In-Service Assessment: *Can be purchased at* <https://www.ebu.com/webshop/>
- Github: <https://github.com/marjohe/UroBot>

**Prompt used for benchmarking:**

**UroBot prompt:**

updated_query = "You are answering questions of an exam, you must reply only with the character of the correct answer. " \

f"You are giving an answer based on the following context: \n" \

f"---" \

f"{context}" \

f"--- \n"

**GPT-based model prompt:**

updated_query = "You are answering questions of an exam," \

" you must reply only with the character of the correct answer, for example 'D', or 'A'."
