# Supplementary material for: Superhuman performance on urology board questions using an explainable language model enhanced with European Association of Urology guidelines
Source: ESMO Real World Data Digit Oncol. 2024 Oct 4;6:100078. doi: 10.1016/j.esmorw.2024.100078 (PMC12836625; doi:10.1016/j.esmorw.2024.100078)
Supplement: Supplementary Methods [file mmc2.docx]

**Checklist for supervised clinical ML study**

| **Before paper submission** | | | |  |
| --- | --- | --- | --- | --- |
| **Study design (Part 1)** | **Completed:**  **page number** | | **Notes if not completed** |  |
| The clinical problem in which the model will be employed is clearly detailed in the paper. | x | 1-3 |  |  |
| The research question is clearly stated. | x | 3 |  |  |
| The characteristics of the cohorts (training and test sets) are detailed in the text. | ☐ |  | not applicable |  |
| The cohorts (training and test sets) are shown to be representative of real-world clinical settings. | ☐ |  | not applicable |  |
| The state-of-the-art solution used as a baseline for comparison has been identified and detailed. | x | 5 |  |  |
| **Data and optimization (Parts 2, 3)** | **Completed:**  **page number** | | **Notes if not completed** |  |
| The origin of the data is described and the original format is detailed in the paper. | x | 4 |  |  |
| Transformations of the data before it is applied to the proposed model are described. | ☐ |  | not applicable |  |
| The independence between training and test sets has been proven in the paper. | ☐ |  | not applicable |  |
| Details on the models that were evaluated and the code developed to select the best model are provided. | x | 4 |  |  |
|  |  |  |  |  |
| Is the input data type structured or unstructured? | ☐ Structured x Unstructured | | |  |
| **Model performance (Part 4)** | **Completed:**  **page number** | | **Notes if not completed** |  |
| The primary metric selected to evaluate algorithm performance (eg: AUC, F-score, etc) including the justification for selection, has been clearly stated. | x | 8 |  |  |
| The primary metric selected to evaluate the clinical utility of the model (eg PPV, NNT, etc) including the justification for selection, has been clearly stated. | x | 8 |  |  |
| The performance comparison between baseline and proposed model is presented with the appropriate statistical significance. | x | 9-11 |  |  |
| **Model Examination (Parts 5)** | **Completed:**  **page number** | | **Notes if not completed** |  |
| Examination Technique 1^a^ | ☐ |  | not applicable |  |
| Examination Technique 2^a^ | ☐ |  | not applicable |  |
| A discussion of the relevance of the examination results with respect to model/algorithm performance is presented. | x | 12-15 |  |  |
| A discussion of the feasibility and significance of model interpretability at the case level if examination methods are uninterpretable is presented. | x | 7,12 |  |  |
| A discussion of the reliability and robustness of the model as the underlying data distribution shifts is included. | x | 12 |  |  |
| *Common examination approaches based on study type:  * For studies involving exclusively structured data coefficients and sensitivity analysis are often appropriate  * For studies involving unstructured data in the domains of image analysis or NLP: saliency maps (or equivalents) and sensitivity analysis are often appropriate |  |  | not applicable |  |
| **Reproducibility (Part 6): choose appropriate tier of transparency** | | | **Notes** |  |
| Tier 1: complete sharing of the code | | x | 4, [*https://github.com/DBO-DKFZ/UroBot*](https://github.com/DBO-DKFZ/UroBot) |  |
| Tier 2: allow a third party to evaluate the code for accuracy/fairness; share the results of this evaluation | | ☐ |  |  |
| Tier 3: release of a virtual machine (binary) for running the code on new data without sharing its details | | ☐ |  |  |
| Tier 4: no sharing | | ☐ |  |  |

PPV: Positive Predictive Value

NNT: Numbers Needed to Treat

^a^ Common examination approaches based on study type: for studies involving exclusively structured data, coefficients and sensitivity analysis are often appropriate; for studies involving unstructured data in the domains of image analysis or natural language processing, saliency maps (or equivalents) and sensitivity analyses are often appropriate. Select 2 from this list or chose an appropriate technique, document each technique used on the appropriate line above.
